# Integrative transcriptomics and metabolomics reveal the biosynthesis of flavonoid metabolites in *Tilia miqueliana Maxim.* leaves

**DOI:** 10.3389/fpls.2025.1642949

**Published:** 2025-09-12

**Authors:** Yajing Zhou, Yongbao Shen

**Affiliations:** ^1^ College of Forestry and Grassland, Nanjing Forestry University, Nanjing, China; ^2^ Collaborative Innovation Center of Sustainable Forestry in Southern China, Nanjing Forestry University, Nanjing, China; ^3^ Southern Tree Seed Inspection Center, National Forestry and Grassland Administration, Nanjing, China

**Keywords:** *Tilia miqueliana* Maxim. leaf, secondary metabolites, flavonoids, transcriptomics and metabolomics, WGCNA analysis

## Abstract

*Tilia miqueliana* Maxim. is renowned for its rich bioactive compounds, including flavonoids, phenolic acids, coumarins, and other secondary metabolites, which possess antioxidant, anticancer, antidepressant, and analgesic effects. This study aims to investigate the seasonal dynamic changes of secondary metabolites in *T. miqueliana* leaves and their biosynthetic regulatory mechanisms. The leaves of *T. miqueliana* were sampled at four different growth stages. Total flavonoids, phenolic acids, amino acids, coumarins, and terpenoid contents were determined using UV spectrophotometry, and enzyme activities of phenylalanine ammonia-lyase (PAL), cinnamate-4-hydroxylase (C4H), and 4-coumarate: CoA ligase (4CL) were measured. Flavonoid monomers such as quercetin and kaempferol, along with endogenous hormones, were quantitatively analyzed using high-performance liquid chromatography (HPLC). Widely targeted metabolomic analysis via UPLC-MS/MS and Illumina transcriptomic sequencing identified 1971 metabolites. The results showed that flavonoids, amino acids and their derivatives, and phenolic acids accounted for nearly half of the total metabolites. The major active substances exhibited significant variations across different developmental stages. The summer months (June to August) represented the most active growth and metabolic phase. Active compounds, represented by flavonoids such as tiliroside, scopoletin, naringenin, dihydrokaempferol, apigenin, luteolin, quercetin, kaempferol, and rutin, are secondary metabolites with potential medicinal value in *T. miqueliana* leaves. There were significant differences in differentially accumulated metabolites (DAMs) and differentially expressed genes (DEGs) across developmental stages. The synthesis of key secondary metabolites is co-regulated by endogenous hormones, enzyme activities, and differentially expressed candidate genes. This study provides new insights for determining the appropriate harvesting time for *T. miqueliana* leaves and the metabolic regulation of secondary metabolites.

## 1 Introduction

*Tilia miqueliana* Maxim. is a deciduous tree of the Malvaceae family, mainly distributed in Jiangsu, Zhejiang, Anhui, and Guangdong provinces of China. It holds ecological and economic value, serving as timber, nectar source, ornamental plant, and a source of medicinal compounds. Its flowers, leaves, and buds contain bioactive substances with central nervous system effects, including anticonvulsant, sedative, and analgesic properties (Cardenas-Rodriguez et al., 2014; Allio et al., 2015; Aguirre-Hernandez et al., 2010). Methanol extracts from leaves and flowers are rich in flavonoids such as quercetin, rutin, isoquercitrin, and kaempferol glycosides, which exhibit antioxidant and neuropharmacological activities (Loscalzo et al., 2009). In Europe, linden flower tea has traditionally been used to treat colds, bronchitis, and inflammation, due to its high content of flavonoids, phenolic acids, and coumarins with antioxidant, anti-inflammatory, anticancer, and antibacterial effects (Nenni and Karahuseyin, 2024; Pavlović et al., 2020). Flavonoids in *T. miqueliana* have shown anticancer, antidepressant, and sedative effects, making them promising therapeutic agents (Zhou et al., 2024). Their biosynthesis originates from the phenylalanine pathway, where phenylalanine is converted to cinnamic acid via PAL, followed by sequential reactions involving C4H, 4CL, CHS, and CHI, leading to various flavonoid subtypes such as flavones, flavonols, and isoflavones. These pathways are developmentally regulated and respond to external cues, with key enzymes (CHS, CHI, F3H) controlled by transcription factors like MYB, bHLH, and WD40, which form MYB-bHLH-WD40 complexes (Carvalho Lemos et al., 2019; Yi, 2015; Falcone Ferreyra et al., 2012; Li and Ahammed, 2023).

However, the metabolomic landscape and regulatory mechanisms of flavonoid biosynthesis in *T. miqueliana* across different developmental stages remain poorly understood. This study integrates widely targeted metabolomics and transcriptomics to profile leaf samples at four growth stages, identifying key metabolites and regulatory pathways. The findings enhance understanding of flavonoid accumulation and offer a foundation for optimized use in pharmacological and nutraceutical applications.

## 2 Materials and methods

### 2.1 Plant materials and treatments

**Figure 1:**
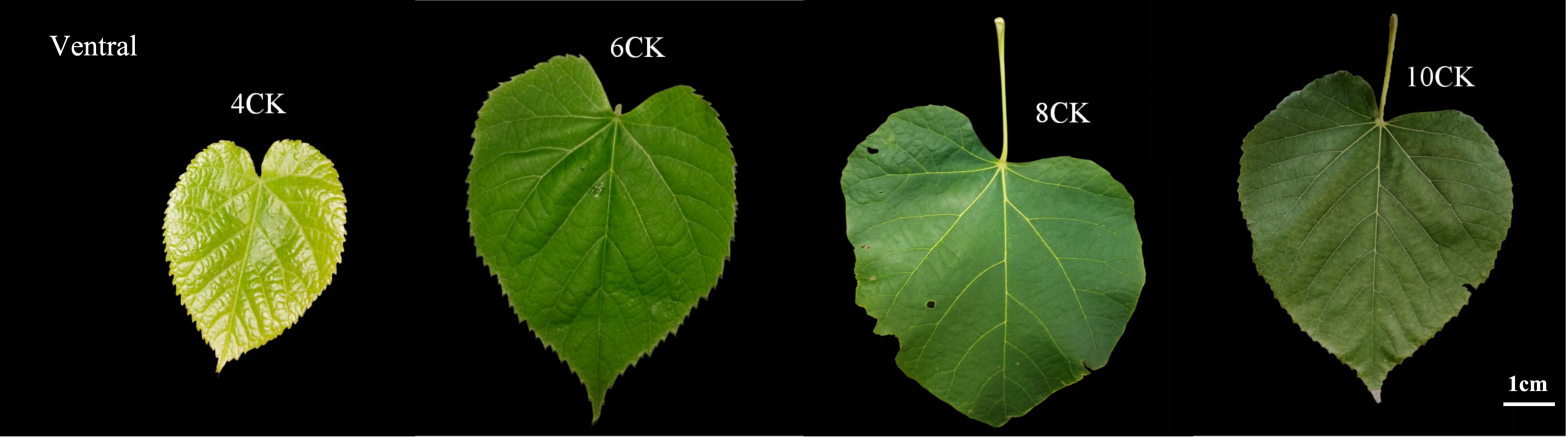
The phenotypes of *Tilia miqueliana* Maxim. leaves during four developmental stages.

Container-grown *Tilia miqueliana* Maxim. seedlings from Nanjing Forestry University (32.06°N, 118.78°E) were exposed to full sunlight outdoors. The experiment included 30 containers (3 replicates, 10 containers per unit). Seedlings were weeded, watered, and fertilized without pesticides, with controlled environmental conditions (temperature, humidity, light duration, and intensity). Sampling occurred in April, June, August, and October, with leaves collected in the middle of each month, labeled as 4CK, 6CK, 8CK, and 10CK (**Figure 1**). One healthy leaf from the middle of each tree was collected, de-veined, cut into pieces, mixed, frozen in liquid nitrogen, and stored at -80°C for RNA extraction.

### 2.2 Widely targeted metabolomics analysis using UPLC-MS/MS

Samples were freeze-dried (Scientz-100F) and ground (MM 400, Retsch). 50 mg of powder was mixed with 1200 μL of -20°C 70% methanolic extract. Vortexed and centrifuged (12000 rpm, 3 min), the supernatant was filtered and stored for UPLC-MS/MS analysis. UPLC conditions: Agilent SB-C18 column (1.8 µm, 2.1 mm × 100 mm), mobile phase: 0.1% formic acid in water (A) and acetonitrile (B). Gradient: 95% A, 5% B to 5% A, 95% B over 9 min, then back to 95% A, 5% B. Flow rate: 0.35 mL/min, column temperature: 40°C, injection volume: 2 μL. MS analysis was performed on an ESI-QTRAP-MS system (https://sciex.com.cn/). ESI parameters: source temperature 550°C, voltage 5500 V (positive)/-4500 V (negative), gases at 50, 60, and 25 psi. MRM scans were used, with collision gas (nitrogen) at medium. MRM transitions were optimized for specific metabolites.

### 2.3 Transcriptome sequencing and analysis

*Tilia miqueliana* Maxim. samples were extracted using ethanol precipitation and CTAB-PBIOZOL. After extraction, RNA was dissolved in 50 µL of DEPC-treated water and quantified using a Qubit fluorometer and Qsep400 biofragment analyzer. cDNA libraries were sequenced on the Illumina platform by Metware Biotechnology Co., Ltd. (Wuhan, China). PolyA mRNAs were enriched using Oligo(dT) magnetic beads, fragmented, and reverse transcribed into first-strand cDNA. Strand-specific second-strand synthesis was performed using dUTPs. After adapter ligation, DNA magnetic bead purification, and fragment selection, a 250–350 bp library was amplified by PCR. The library was then cyclized to obtain single-stranded circular DNA and amplified to generate DNA nanoballs (DNBs), which were loaded into the sequencing chip for sequencing on the BGI platform. Raw data were filtered using fastp to remove adapters and low-quality reads. Clean reads were assembled using Trinity (https://github.com/trinityrnaseq/trinityrnaseq), and redundancy was removed using Corset. CDS prediction was performed with TransDecoder (https://github.com/TransDecoder/), and the amino acid sequences were compared to KEGG, NR, Swiss-Prot, GO, COG/KOG, and TrEMBL databases using DIAMOND BLASTX (Buchfink et al., 2015). The Pfam database was used for further annotation. Transcript expression levels were calculated using RSEM, and FPKM was used to estimate gene expression. Differential expression was analyzed with DESeq2 for biological replicates and edgeR for non-replicates, with criteria of |log2Fold Change| ≥ 1, FDR < 0.05, and Padj ≤ 0.05.

### 2.4 Multivariate analysis of identified metabolites

PCA was performed using the prcomp function in R, with data scaled to unit variance. HCA and Pearson correlation coefficients (PCC) were visualized using heatmaps via the ComplexHeatmap package. Differential metabolites were identified based on VIP > 1 and |Log2FC| ≥ 1.0, extracted from OPLS-DA with 200 permutations to prevent overfitting. Analyses were performed using MetaboAnalystR. Metabolites were annotated with the KEGG Compound database and mapped to pathways in the KEGG Pathway database. Pathway enrichment was analyzed via metabolite set enrichment analysis (MSEA), with significance determined by p-values from the hypergeometric test (Ringnér, 2008; Worley and Powers, 2013; Xia and Wishart, 2010; Kanehisa and Goto, 2000).

### 2.5 Determination of flavonoid, phenolic, coumarin, amino acid content, and enzyme activity

The total flavonoid (TFC), phenolic (TPC), coumarin(TCC), and amino acid (TAAC) contents were quantified using a UV-Vis spectrophotometer(2014) (Da Silva et al., 2015; Nikzad and Parastar, 2021; Núñez et al., 2021; Nor et al., 2022). Rutin, gallic acid, coumarin, and L-arginine were used as reference standards, with results expressed as rutin, gallic acid, coumarin, and L-arginine equivalents (mg/g extract), respectively. The enzyme activities of phenylalanine ammonia-lyase (PAL), cinnamic acid-4-hydroxylase (C4H) and 4-carboxymethyl coenzyme Aligase (4CL) were measured using UV-Vis spectrophotometer (Ge, 1996; Aydaş et al., 2013).

### 2.6 Quantification of quercetin, isorhamnetin, kaempferol, scopoletin, and luteolin

Quercetin, isorhamnetin, kaempferol, scopoletin, and luteolin were measured using a Shimadzu LC-30AD HPLC system with an AB Sciex Qtrap 6500 mass spectrometer. About 0.5 g of *Tilia miqueliana* Maxim. sample was extracted with 5 mL of 75% methanol by ultrasonic extraction (40 min, 250 W, 50 kHz). After filtration through a 0.22 μm membrane, the sample was analyzed. HPLC: Poroshell 120 SB-C18 column (2.1 × 150 mm, 2.7 µm), column temperature 30°C, mobile phase: A = 0.05% formic acid, B = 0.05% formic acid, injection volume 10 µL. MS: ESI negative mode, MRM scan, -4500 V, 650°C. Compound content was calculated as: Content (µg/g) = detected concentration (µg/mL) × extraction volume (mL) ÷ sample mass (g).

### 2.7 Weighted gene co-expression network analysis

This method involves the construction of a weighted adjacency matrix based on pairwise gene expression correlations, followed by its transformation into a Topological Overlap Matrix (TOM) to enhance the robustness of network connectivity. Genes are clustered into modules using hierarchical clustering, and module eigengenes are then correlated with external traits to explore potential biological associations (Li et al., 2024).

### 2.8 Statistical analysis

Statistical analyses were conducted using R software and SPSS statistical software. Analysis of variance (ANOVA) was performed, followed by Duncan’s multiple range test to determine significant differences (p < 0.05). Transcriptional and metabolic data were analyzed and visualized using Metware Cloud (https://cloud.metware.cn), while line graphs were generated using GraphPad Prism 9 (GraphPad Prism 9, San Diego, California, USA) (Li et al., 2021).

## 3 Results

### 3.1 Detection of metabolites and multivariate analysis in leaves of *Tilia miqueliana* Maxim.

**Figure 2:**
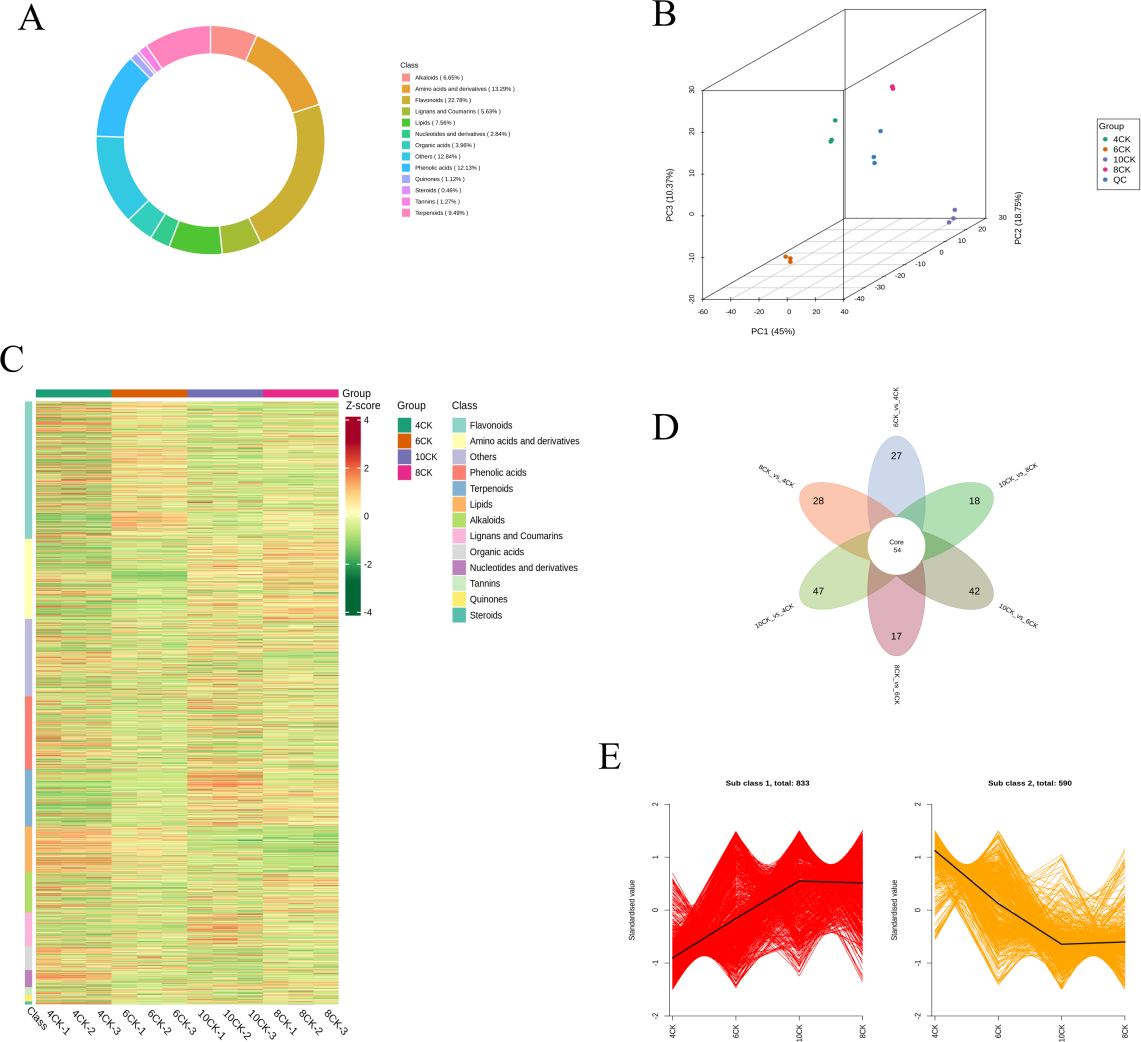
**(A)** Circular plot of metabolite category composition across the four growth stages. **(B)** PCA 3D results plot, including QC samples. **(C)** Heatmap of metabolite contents across the four growth stages, showing categories by metabolite classification. **(D)** Venn diagram of differential groups between various comparisons. **(E)** K-Means clustering trend chart of differential metabolites.

To thoroughly identify and characterize the metabolites in *Tilia miqueliana* Maxim. leaves, we performed UPLC-MS/MS analysis on leaf samples from seedlings at four distinct growth stages: 4CK, 6CK, 8CK, and 10CK. Across these developmental stages, a total of 1,971 unique metabolites were annotated. Among these, we identified 449 flavonoids (22.7%), 262 amino acids and their derivatives (13.29%), 239 phenolic acids (12.13%), 186 terpenoids (9.49%), 149 lipids (7.56%), 131 alkaloids (6.65%), 111 lignans and coumarins (5.63%), 78 organic acids (3.96%), 56 nucleotides and their derivatives (2.84%), 22 quinones (1.12%), 25 tannins (1.27%), 9 steroids (0.46%), and 254 other compounds (12.84%) (**Figure 2A**). Flavonoids, amino acids and their derivatives, along with phenolic acids, together account for nearly half of the total identified metabolites. Principal Component Analysis (PCA) was employed to elucidate the intrinsic structure of multiple variables through the creation of several principal components (Zhong et al., 2024). As shown in **Figure 2B**, the 3D principal component analysis (PCA) effectively distinguishes samples from the four developmental stages, with clear separation along the principal components. PC1, PC2, and PC3 explain 45.00%, 18.75%, and 10.37% of the total variance, respectively, with the first two components accounting for 63.75%. The clustering of biological replicates within each group, along with the central positioning of the quality control (QC) samples, demonstrates high experimental reproducibility and data reliability. Comprehensive metabolomic profiling revealed pronounced stage-specific differences in metabolite composition during leaf development in *Tilia miqueliana* Maxim. Among the four developmental stages, the 4CK group (April) exhibited the most distinct metabolic profile, while the 8CK (August) and 10CK (October) groups demonstrated greater compositional similarity. Hierarchical clustering analysis (**Figure 2C**) effectively distinguished the samples into four discrete clusters, underscoring dynamic metabolic reprogramming across growth stages. Young leaves in April (4CK) were characterized by elevated levels of lipids, amino acids, nucleosides, peptides, carbohydrates, fatty acids, organic acids, flavonoids, and phenolic compounds. Notably, flavonoid and phenolic compound concentrations were highest during the early stages (April and June), but progressively declined with leaf maturation. In contrast, the accumulation of terpenoids, steroids, and certain acids peaked in the late stage (October), while amino acid levels were relatively higher in August and October than in earlier stages.

Differential metabolite analysis (**Figure 2D**) identified 54 core metabolites across all developmental stages. The greatest number of differential metabolites was observed between 10CK and 4CK (47 metabolites), followed by 10CK vs. 6CK (42), 10CK vs. 8CK (18), and 8CK vs. 6CK (17), indicating substantial metabolic shifts throughout leaf development. K-means clustering of differential metabolites (**Figure 2E**) revealed two dominant expression patterns. Subclass 1, comprising 833 metabolites, exhibited a general upregulation trend, with minimal accumulation at 4CK, peaking at 10CK, and a slight decline at 8CK, suggesting activation of specific metabolic pathways during maturation. Conversely, Subclass 2, containing 590 metabolites, displayed a continuous downregulation trend, with the highest levels at 4CK and gradual decline thereafter. This pattern may reflect the repression of certain biosynthetic pathways or enhanced metabolic stress during later stages of development.

### 3.2 Screening for DAM based on top fold change distribution compounds

We performed a comparative analysis between growth stages (6CK vs. 4CK, 8CK vs. 4CK, 10CK vs. 4CK, 6CK vs. 8CK, 6CK vs. 10CK, and 8CK vs. 10CK) using the supervised OPLS-DA technique to assess metabolite differences (**Supplementary Figure S1**). The analysis revealed clear separation between groups, indicating significant metabolic differences across the stages. Differentially accumulated metabolites (DAMs) were identified from all detected metabolites (**Supplementary Table S1**), and results were visualized using volcano plots (**Supplementary Figure S2A–F).** A total of 759 DAMs were identified between 6CK and 4CK, with 449 upregulated and 310 downregulated. Between 8CK and 4CK, 1045 DAMs were identified, and 666 DAMs were found between 8CK and 6CK. A total of 1059 DAMs were identified between 10CK and 4CK, and the fewest DAMs (371) were found between 10CK and 8CK, with 192 upregulated and 179 downregulated. Detailed data can be found in **Supplementary Table S2**.

**Figure 3:**
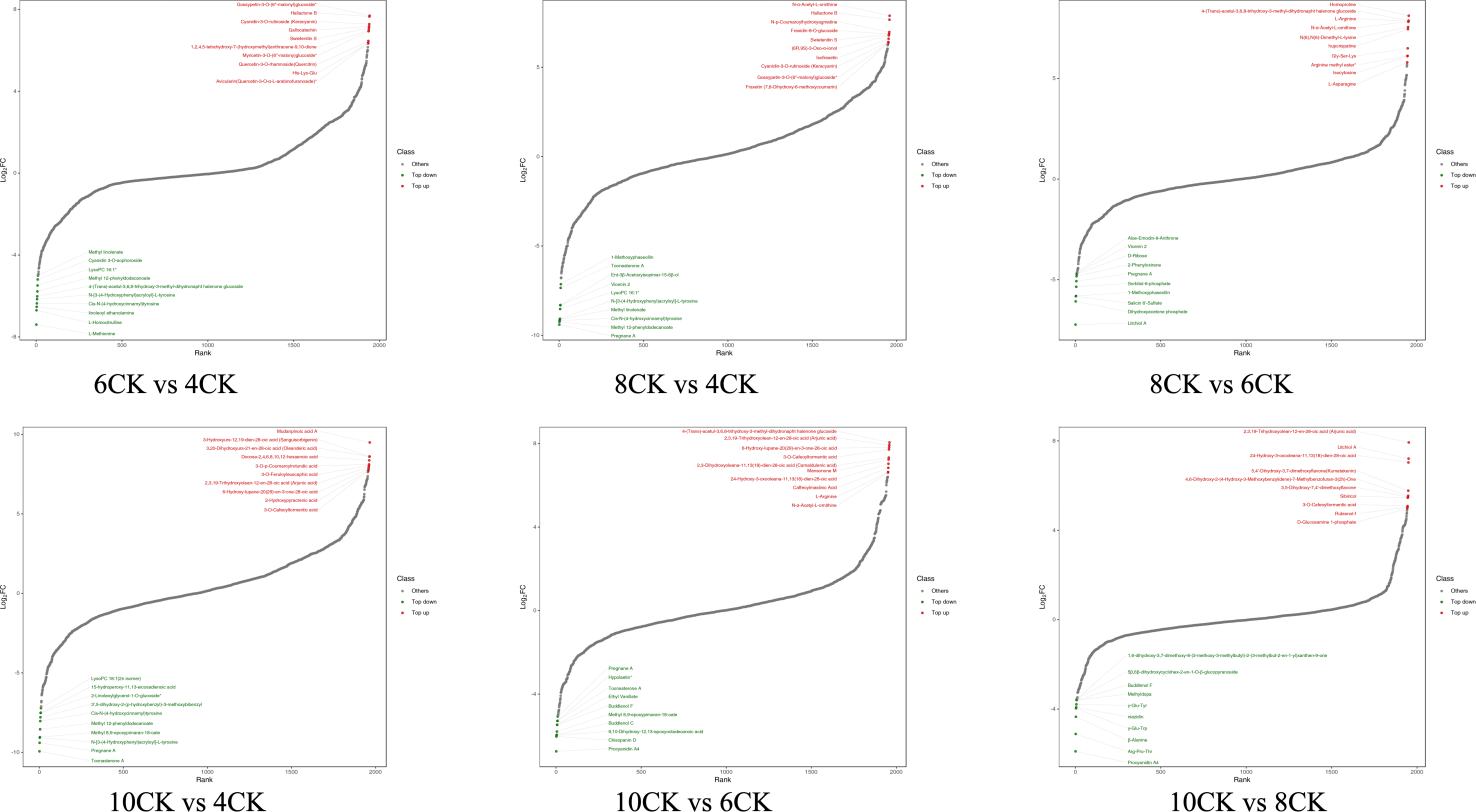
The dynamic distribution map of the top 10 upregulated and downregulated differential metabolites.

Differential metabolite trend analysis revealed distinct metabolic patterns across the comparisons (**Figure 3**). In the 6CK vs. 4CK comparison, metabolites such as Gossypetin, Keracyanin, and Hallactone were upregulated, while Methyl linolenate, Cyanidin 3-O-sophoroside, and Tyrosine were downregulated. In the 8CK vs. 4CK comparison, Hallactone, Fraxidin, and Swietenitin were upregulated, while Methoxphaseollin, Toonasterone A, and Tyrosine were downregulated. In the 10CK vs. 4CK comparison, Mudanpinoic acid A, Sanguisorbigenin, and Arjunic acid were elevated, while 2n isomer, Tyrosine, and Pregnane A were reduced. In the 8CK vs. 6CK comparison, Homoproline, L-Arginine, and Hupcrispatine were upregulated, while Aloe-Emodin-9-Anthrone, Vicenin 2, and Pregnane A were downregulated. In the 10CK vs. 6CK comparison, Arjunic acid, Camaldulenic acid, and L-Arginine were upregulated, while Pregnane A, Toonasterone A, and Procyanidin A4 were downregulated. Finally, in the 10CK vs. 8CK comparison, Arjunic acid, Litchiol A, and Rubianol-f were upregulated, while Buddlenol F, Methyldopa, and Procyanidin A4 were downregulated.

### 3.3 RNA-seq and KEGG enrichment analysis across four growth stages

**Figure 4:**
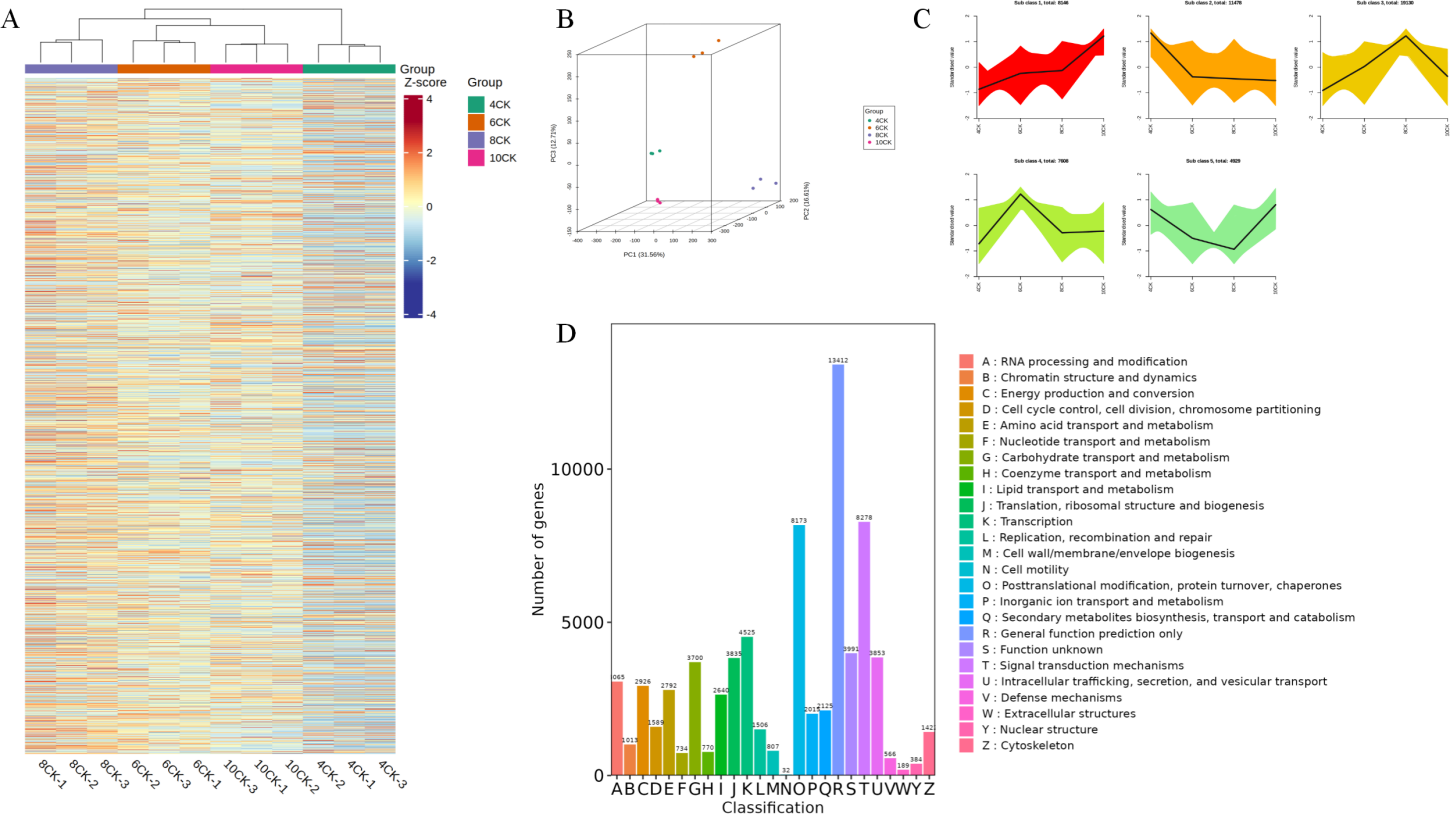
**(A)** All DEG heatmap. **(B)** 3D PCA analysis of unigene expression. **(C)** K-means cluster. **(D)** Unigene cluster of orthologous groups(KOG) classification.

To investigate gene expression changes at the transcriptional level during different growth stages of *Tilia miqueliana* Maxim. leaves, RNA-seq analysis was conducted on 12 samples, generating a total of 77.87 Gb of clean data, with each sample achieving at least 5 Gb. All samples had a Q30 base percentage above 94%. The clustering heatmap of differentially expressed genes demonstrated good reproducibility across the four sample groups (**Figure 4A**). In the 4CK group, certain metabolites exhibited lower Z-scores (blue), while in the 8CK group, some metabolites showed higher Z-scores (red). Principal component analysis (PCA) revealed distinct separation between the sample groups, with PC1 explaining 45% of the total variance, PC2 18.75%, and PC3 10.37% (**Figure 4B**). K-means clustering grouped genes with similar expression patterns into five major clusters (**Figure 4C**). Gene functional annotation classified 13,412 genes under General function prediction only, while 8,173 genes were categorized into Posttranslational modification, protein turnover, and chaperones, and 8,278 genes into “Signal transduction mechanisms” (**Figure 4D**).

**Figure 5:**
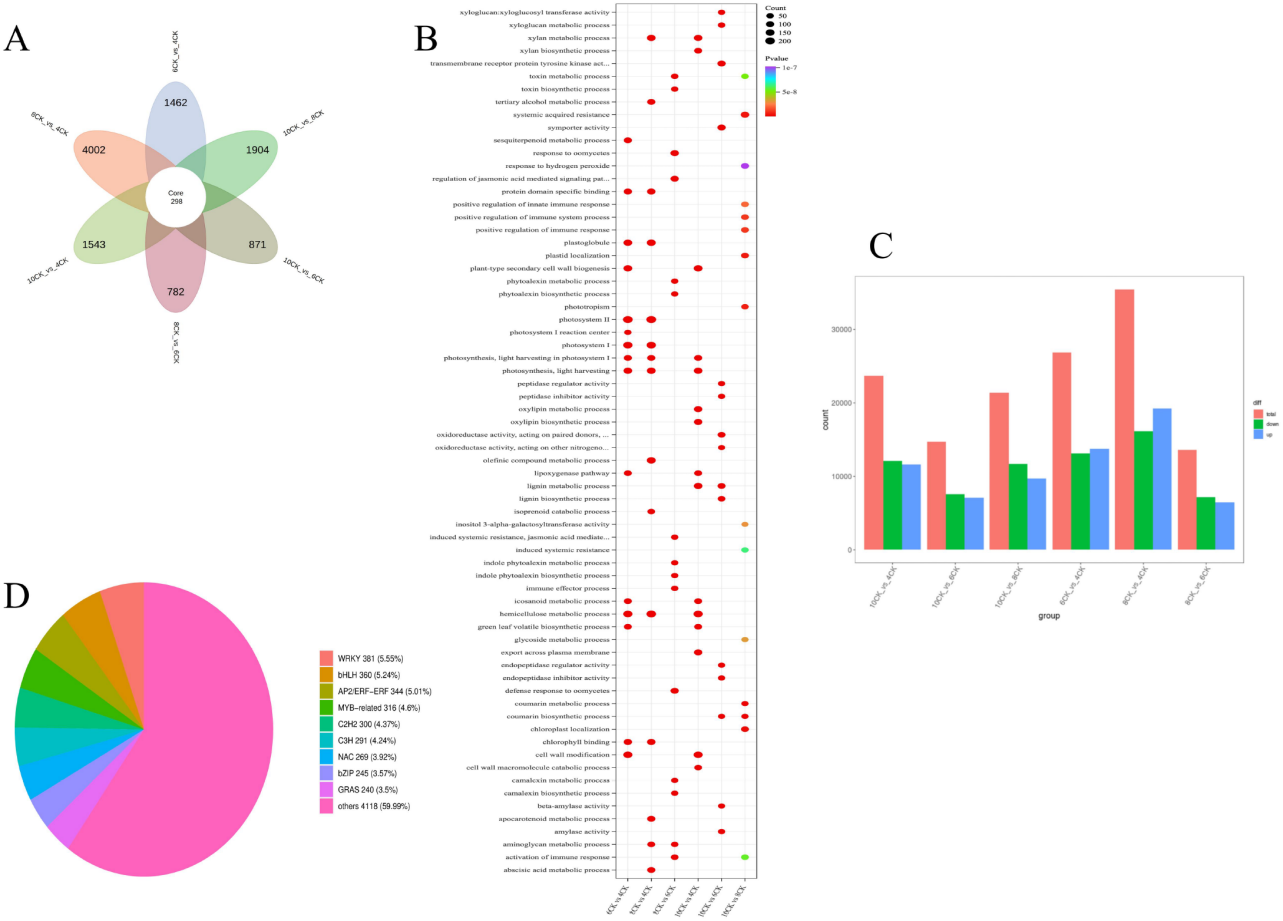
**(A)** Pairwise comparison of differentially expressed gene (DEG) petal diagram. **(B)** Pairwise comparison GO enrichment bubble plot. **(C)** Bar chart of upregulated and downregulated genes in pairwise comparisons. **(D)** Pie chart of transcription factor distribution among all genes.

The pairwise comparison petal diagram (**Figure 5A**) shows 298 core differentially expressed genes (DEGs). The largest number of DEGs was observed in the 8CK vs. 4CK group (4,002), followed by the 10CK vs. 8CK group (1,904) and 10CK vs. 4CK group (1,543), while the 10CK vs. 6CK group had the fewest (871). GO enrichment analysis (**Figure 5B**) identified significant enrichment in terms related to lignin metabolism, photosynthesis (photosystem I & II), immune response activation, and other metabolic processes. Particularly, photosynthesis-related genes were enriched across multiple comparisons, indicating their involvement in developmental changes. Immune response-related terms were also enriched, suggesting changes in immune regulation at different stages of leaf development. Lignin biosynthesis terms were enriched in several comparisons, indicating their role in secondary metabolism or stress response in leaves.

A total of 148,737 genes were detected, with 51,291 identified as DEGs, based on the criteria of |log2 FC| ≥ 1 and FDR < 0.05. DEGs across comparisons include: 26,886 between 6CK and 4CK (13,139 downregulated, 13,747 upregulated), 35,464 between 8CK and 4CK (16,178 downregulated, 19,286 upregulated), 13,645 between 8CK and 6CK (7,191 downregulated, 6,454 upregulated), 23,720 between 10CK and 4CK (12,092 downregulated, 11,628 upregulated), 14,687 between 10CK and 6CK (7,560 downregulated, 7,127 upregulated), and 21,394 between 10CK and 8CK (11,667 downregulated, 9,727 upregulated) (**Figure 5C**). KEGG enrichment analysis of DEGs and DAMs identified the top 25 enriched pathways (**Supplementary Figure S3**). Transcription factors were largely classified into the WRKY, bHLH, MYB-related, and AP2/ERF families (**Figure 5D**).

**Figure 6:**
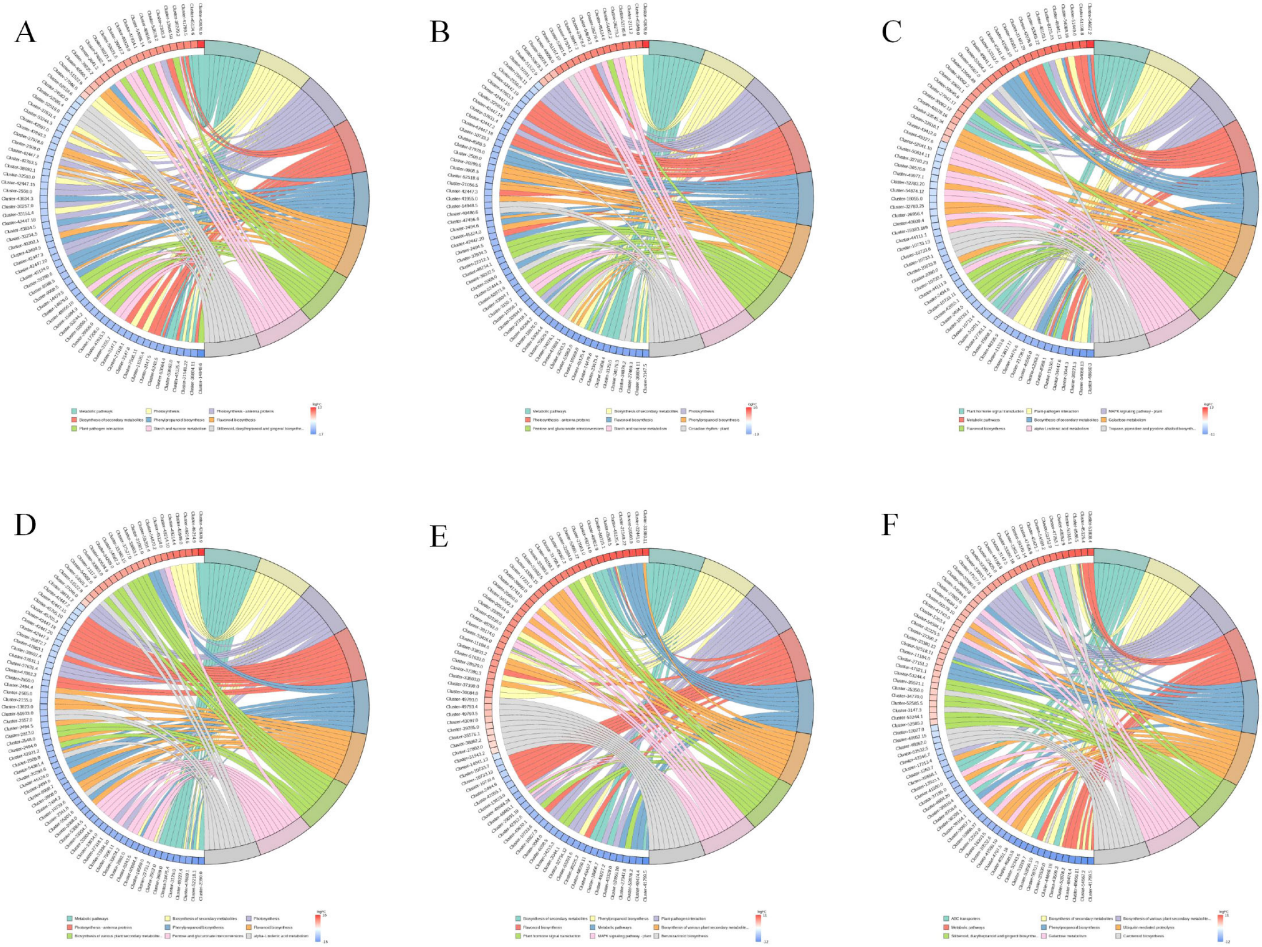
GO Enrichment chordplot. **(A)** 6CK vs 4CK, **(B)** 8CK vs 4CK, **(C)** 10CK vs 4CK, **(D)** 8CK vs 6CK, **(E)** 10CK vs 6CK, and **(F)** 10CK vs 8CK.

**Figure 7:**
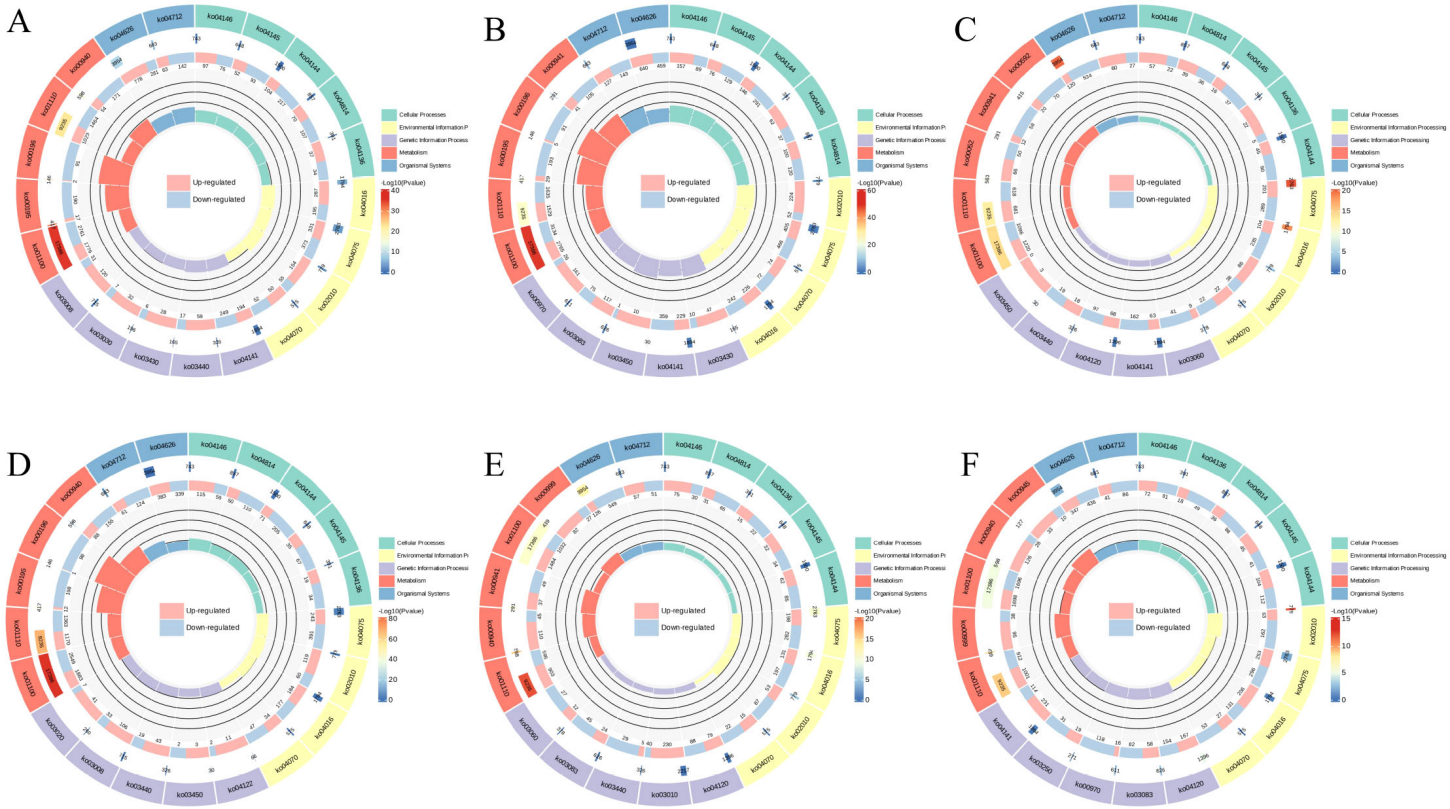
GO Enrichment circos. **(A)** 6CK vs 4CK, **(B)** 8CK vs 4CK, **(C)** 10CK vs 4CK, **(D)** 8CK vs 6CK, **(E)** 10CK vs 6CK, and **(F)** 10CK vs 8CK.

The chord diagram (**Figure 6**) shows the complex relationships between different gene clusters and associated biological functions, revealing key pathways involved in photosynthesis, metabolic processes, and potentially other cellular functions. The circular plot (**Figure 7**) visualizes the results of a GO enrichment analysis, highlighting the significant biological processes, cellular components, and molecular functions enriched in the up-regulated and down-regulated gene sets.

**Figure 8:**
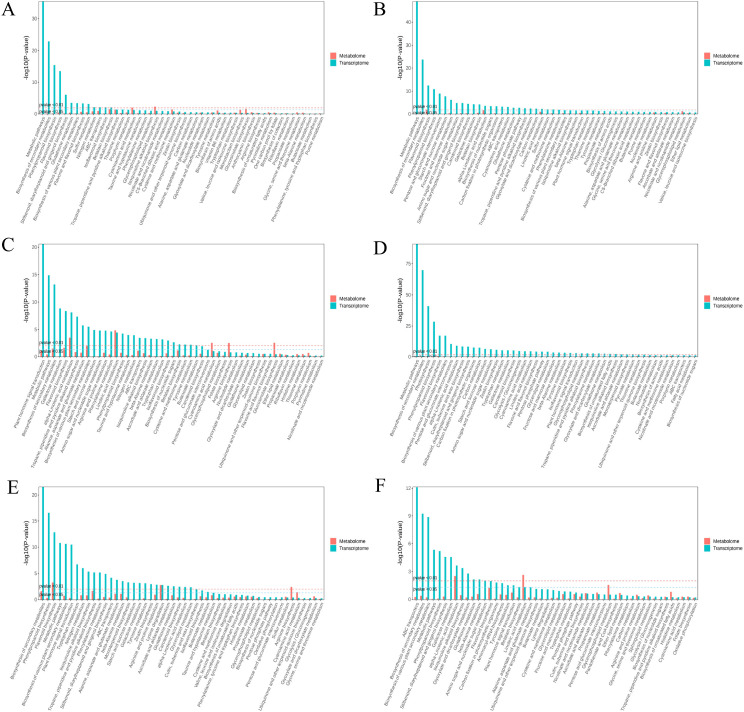
KEGG enrichment analysis for metabolome and transcriptome: **(A)** 6CK vs. 4CK, **(B)** 8CK vs. 4CK, **(C)** 8CK vs. 6CK, **(D)** 10CK vs. 4CK, **(E)** 10CK vs. 6CK, and **(F)** 10CK vs. 8CK.

In the transcriptome analysis, the 6CK vs. 4CK, 8CK vs. 4CK, and 10CK vs. 4CK comparisons were enriched in metabolic pathways and secondary metabolite biosynthesis (**Figure 8**). The 8CK vs. 6CK pathways focused on plant hormone signal transduction and metabolism. For the metabolome, enriched pathways included linoleic acid metabolism, cyanoamino acid metabolism, and arginine biosynthesis. In the 10CK vs. 6CK comparison, transcriptome pathways were enriched in secondary metabolite and phenylpropanoid biosynthesis, while metabolome pathways involved arginine biosynthesis and lysine degradation. The 10CK vs. 8CK comparison showed enrichment in ABC transporters and secondary metabolite biosynthesis at the transcriptome level, and linoleic acid metabolism and glycerophospholipid metabolism in the metabolome. Unigene pathways are listed in **Supplementary Table S4**.

### 3.4 The key bioactive metabolites and enzyme activities in *Tilia miqueliana* Maxim.

**Figure 9:**
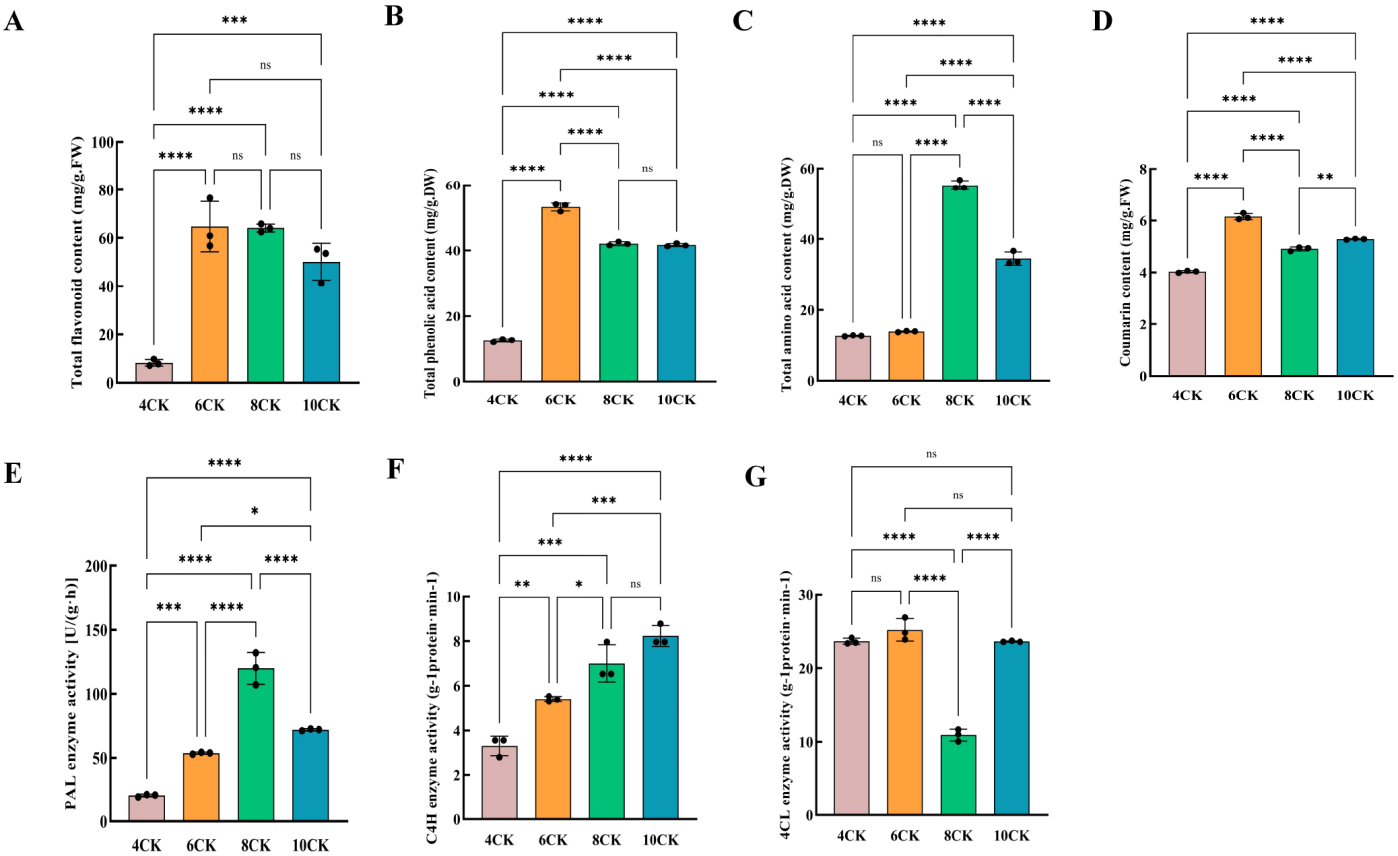
**(A)** Total flavonoid content, **(B)** Total phenolic content, **(C)** Total amino acid content, **(D)** Total coumarin content, **(E)** PAL enzyme activity, **(F)** C4H enzyme activity, **(G)** 4CL enzyme activity. #:* (one asterisk): Indicates a statistically significant difference with a p-value between 0.01 and 0.05. p < 0.05. ** (two asterisks): Indicates a statistically significant difference with a p-value between 0.001 and 0.01. p < 0.01. *** (three asterisks): Indicates a highly statistically significant difference with a p-value between 0.0001 and 0.001. p < 0.001. **** (four asterisks): Indicates an extremely statistically significant difference with a p-value less than 0.0001. p < 0.0001.

In *Tilia miqueliana* Maxim. leaves, flavonoids were the most abundant bioactive compounds, followed by phenolic acids, coumarins, and other substances. Key flavonoids, including catechin, hesperetin, and quercetin, along with lignans (syringaresinol) and triterpenoids (corosolic acid), were identified. Tiliroside and resveratrol, known for their antidepressant and anticancer properties, were first detected in these leaves (**Supplementary Table S3**, **Supplementary Figure S4**). Flavonoid content was lowest in April and highest in June and August, with a decrease in October (**Figure 9A**). Phenolic acid peaked in June and was lowest in April (**Figure 9B**). Total amino acids increased in August and decreased in October (**Figure 9C**). Coumarin levels were highest in June, with significant variation between months (**Figure 9D**). Enzymes involved in the phenylalanine pathway—PAL, C4H, and 4CL—showed varying activity across months. PAL activity peaked in August (**Figure 9E**), C4H steadily increased, with the highest activity in October (**Figure 9F**), while 4CL activity was lowest in August (**Figure 9G**). These enzymes drive the flavonoid biosynthesis pathway.

**Figure 10:**
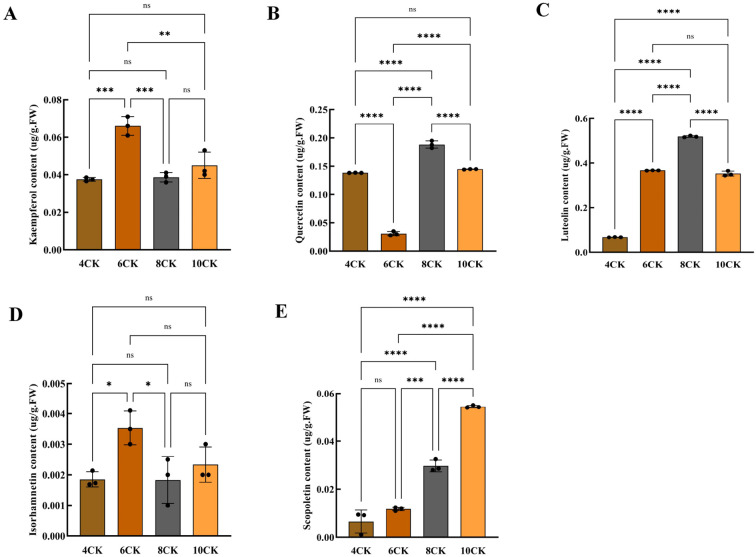
**(A)** Kaempferol content, **(B)** Quercetin content, **(C)** Luteolin content, **(D)** Isorhamnetin content, **(E)** Scopoletin content. #:* (one asterisk): Indicates a statistically significant difference with a p-value between 0.01 and 0.05. p < 0.05. ** (two asterisks): Indicates a statistically significant difference with a p-value between 0.001 and 0.01. p < 0.01. *** (three asterisks): Indicates a highly statistically significant difference with a p-value between 0.0001 and 0.001. p < 0.001. **** (four asterisks): Indicates an extremely statistically significant difference with a p-value less than 0.0001. p < 0.0001.

Kaempferol, quercetin, luteolin, isorhamnetin, and scopoletin are the main active compounds contributing to the pharmacological effects in *Tilia miqueliana* Maxim. leaves (Du et al., 2009). Kaempferol(0.06 µg/g) and isorhamnetin (0.003 µg/g) were highest in June (**Figure 10A**). Quercetin (0.182 µg/g) exhibited the highest content in August, with values of 0.138 µg/g and 0.145 µg/g observed in April and October, respectively. The lowest content was recorded in June (**Figure 10B**). Luteolin content was lowest in April(0.0682 µg/g) and highest in August(0.516 µg/g), with significant differences observed across all months (p<0.0001), but no difference between June and October (**Figure 10C**). Isorhamnetin content in June was significantly higher than in April and August (p<0.05), with no significant differences between other months (**Figure 10D**). Scopoletin content increased progressively from April to October, with no significant difference between April and June. August showed higher levels than June (p<0.001), and October (0.0536 µg/g) had the highest content compared to all other months (p<0.0001) (**Figure 10E**).

**Figure 11:**
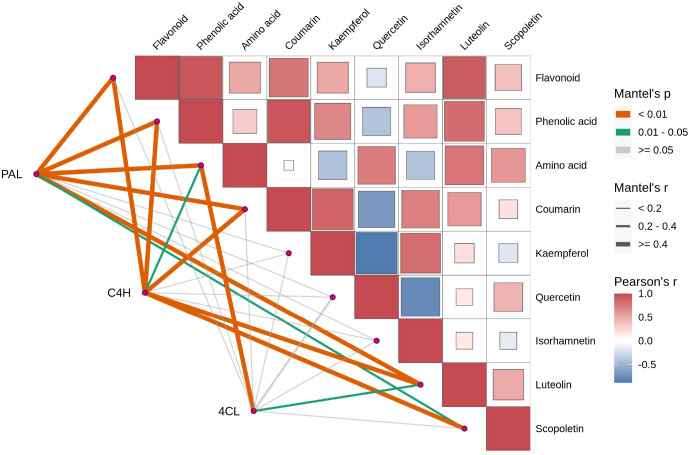
Mantel Test and Pearson correlation coefficient analysis of the relationships between various compounds (flavonoid, phenolic acid, amino acid, coumarin, kaempferol, quercetin, isorhamnetin, luteolin, scopoletin) and the enzymes PAL, C4H, and 4CL. Metabolic modules with significant Mantel correlations (p < 0.05 and r ≥ 0.2) with key enzymes should be prioritized. In the Pearson’s r heatmap, red denotes positive correlations, with darker shades indicating stronger correlations, while blue represents negative correlations, with darker shades indicating stronger negative correlations. White represents no significant or near-zero correlation. In the Mantel test, orange p-values (< 0.01) indicate highly significant correlations, green p-values (0.01-0.05) indicate significant correlations, and gray p-values (> 0.05) indicate non-significant correlations. Line thickness reflects Mantel’s r: thick lines (r ≥ 0.4) indicate strong correlations, medium lines (r = 0.2-0.4) indicate moderate correlations, and thin lines (r < 0.2) indicate weak correlations.

The correlations between the enzymes PAL, C4H, and 4CL and various compounds in *Tilia miqueliana* Maxim. leaves exhibit distinct and significant patterns. Both PAL and C4H demonstrate strong, highly significant correlations with flavonoids and phenolic acids (p<0.01, r>0.4), while 4CL shows weak or non-significant correlations with these metabolites (p>0.05, r<0.2). In the case of amino acids, PAL and 4CL exhibit strong, highly significant correlations (p<0.01, r>0.4), whereas C4H displays a moderate but significant correlation (0.01<p<0.05, 0.2<r<0.4). Similarly, PAL and C4H are strongly correlated with coumarins (p<0.01, r>0.4), while 4CL shows a weak, non-significant correlation (p>0.05, r<0.2). Regarding specific flavonoids, all three enzymes exhibit weak or non-significant correlations with kaempferol, quercetin, and isorhamnetin (p>0.05, r<0.2). Notably, PAL and C4H show strong, highly significant correlations with luteolin (p<0.01, r>0.4), while 4CL presents a moderate but significant correlation (0.01<p<0.05, 0.2<r<0.4). For scopoletin, C4H exhibits a strong, highly significant correlation (p<0.01, r>0.4), PAL shows a moderate but significant correlation (0.01<p<0.05, 0.2<r<0.4), and 4CL demonstrates a weak, non-significant correlation (p>0.05, r<0.2) (**Figure 11**).

### 3.5 WGCNA Analysis of piceid, coumarins, and flavonoids

**Figure 12:**
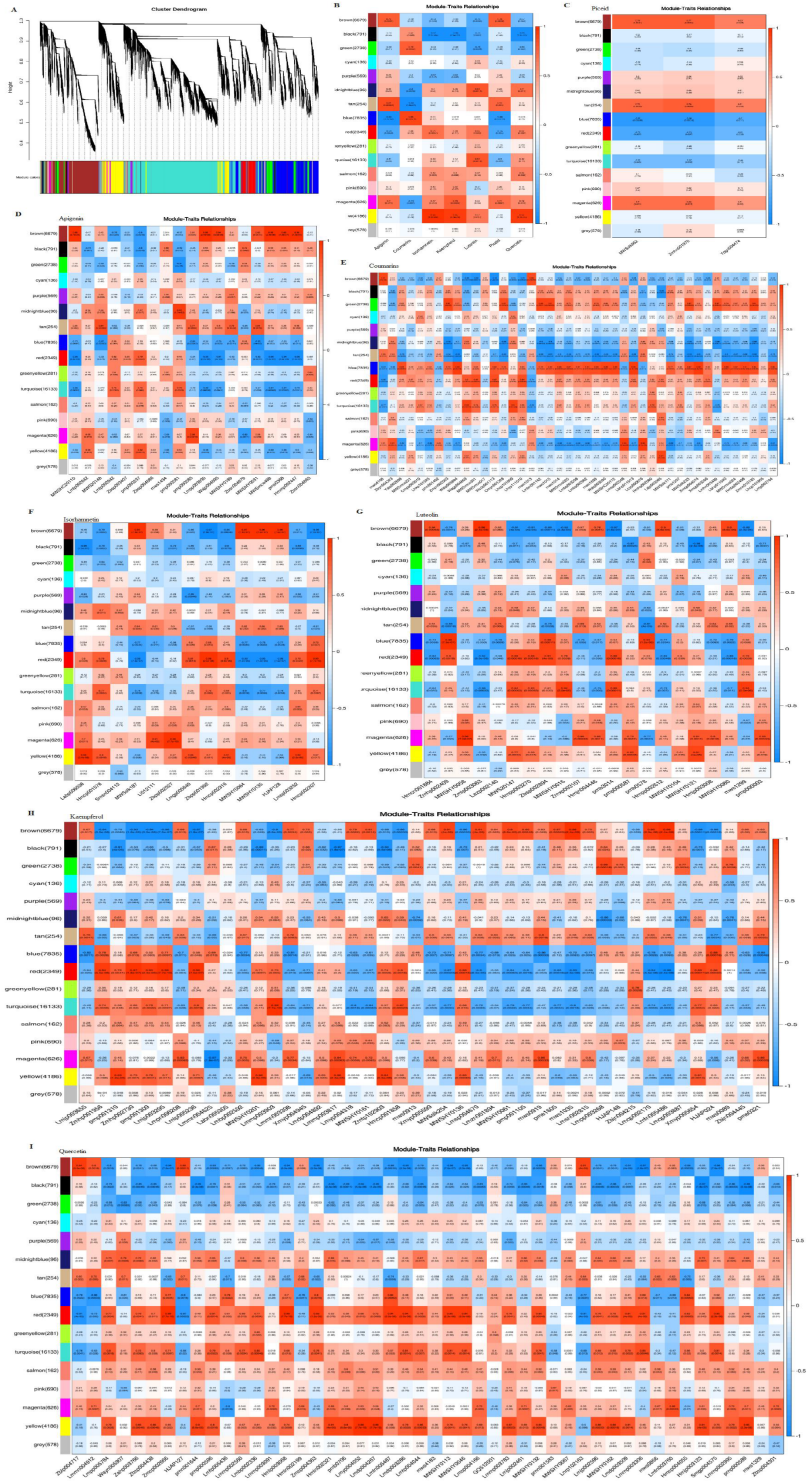
Correlation of seven major bioactive metabolites with WGCNA gene modules: **(A)** Hierarchical cluster dendrogram of 16 expression modules. **(B)** Relationship analysis of the seven major bioactive metabolites. **(C)** Module-piceid relationship analysis. **(D)** Module-apigenin relationship analysis. **(E)** Module-coumarin relationship analysis. **(F)** Module-isorhamnetin relationship analysis. **(G)** Module-luteolin relationship analysis. **(H)** Module-kaempferol relationship analysis. **(I)** Module-quercetin relationship analysis. Each box contains values representing the correlation and significance between the module and the metabolites, with the numbers in each cell indicating the correlation coefficient (r), and the numbers in parentheses representing the p-value. Red indicates a positive correlation, while blue represents a negative correlation. The intensity of the color reflects the strength of the correlation, with darker colors indicating stronger correlations.

To explore circadian relationships between the transcriptome and metabolome, we constructed a co-expression network using WGCNA, correlating 3 piceids, 43 coumarins, and 146 flavonoids (including 19 apigenins, 21 luteolins, 14 isorhamnetins, 44 kaempferols, and 48 quercetins) across different growth stages. Sixteen distinct modules were identified, labeled as brown, black, green, cyan, purple, midnight blue, tan, blue, red, green-yellow, turquoise, salmon, pink, magenta, yellow, and grey (**Figure 12**). Piceid was positively correlated with the brown, tan, and magenta modules, and negatively correlated with the blue, red, and turquoise modules. Coumarins showed positive correlations with the blue, red, and green modules, and negative correlations with the tan, midnight blue, and magenta modules. Apigenin strongly correlated with the brown, tan, and magenta modules, especially the brown module, which was positively correlated with specific apigenin derivatives, such as 8-Methoxyapigenin (MWSHC20110), Apigenin-7-O-(6’’-p-Coumaryl)glucoside (Lmpp003930), Apigenin-7-O-Gentiobioside (Wajp004095), Apigenin-7-O-neohesperidoside (Rhoifolin) (MWSmce498), Apigenin-7-O-rutinoside (Isorhoifolin) (pme0368), and Apigenin-7-O-rutinoside-4’-O-rhamnoside (Hmmp002447) (**Supplementary Figure S5**). Isorhamnetins, kaempferols, and quercetins had the strongest positive correlations with the yellow module and negative correlations with the black module. Flavonoids were positively correlated with the yellow, magenta, pink, salmon, turquoise, red, and midnight blue modules, but negatively correlated with the brown, black, green, and purple modules. The black and green modules were negatively correlated with all metabolites except coumarins, whereas the midnight blue and magenta modules showed the opposite trend (Cui et al., 2024).

## 4 Discussion

### 4.1 Bioactive compounds in leaves of *Tilia miqueliana* Maxim.

Studies have shown that secondary metabolites in Tilia species, including flavonoids, phenolic compounds, and terpenoids, exhibit significant variability depending on developmental stages and environmental factors (Borghi and Fernie, 2021; Bao and Shen, 2022; Cai et al., 2023). For instance, the flavonoid profiles in *Tilia miqueliana* Maxim., *Tilia cordata*, *Tilia amurensis*, and *Tilia tomentosa* are influenced by growth stage, with distinct variations observed in the leaves, bracts, and flowers of these species. Similarly, flavonoid and phenolic compound content in other species, such as *Picea abies* (Pinaceae) and *Olea europaea*, exhibit seasonal fluctuations (Kabbash et al., 2023). In *Tilia miqueliana* Maxim., flavonoids, particularly flavonols, show marked seasonal changes, with peak concentrations of compounds like Naringenin-7-O-glucoside in early spring (April) and quercetin-3-O-galactoside in mid-summer (June). The overall flavonoid content, including catechins and gallocatechins, is highest during the warmer months, with a notable increase in August. These trends are similar to those observed in tea plants, where the composition of flavonoids, including flavonols, varies depending on the harvest season (Liu et al., 2023). In *Actinidia valvata*, kaempferol glycosides are most abundant in June, while other flavonoids peak in October, with isorhamnetin glycosides rising sharply from September to October (Du et al., 2016). The phenolic content in *Tilia miqueliana* Maxim. also peaks in June, consistent with seasonal trends in tea leaves (Zhu et al., 2020).

Coumarins and isoflavones also follow seasonal patterns, with *Tilia miqueliana* Maxim. showing an increase in these compounds during late summer, likely in response to heightened environmental stress, such as increased light intensity and water evaporation. The upregulation of key enzyme genes like PAL, CHI, and F3H under these conditions contributes to the biosynthesis of these secondary metabolites, which play a role in plant defense mechanisms (Ali, 2013). These compounds, such as kaempferol and quercetin, possess antimicrobial and anti-inflammatory properties, and also act as signaling molecules regulating stress responses in plants (Jan et al., 2022). In vegetables, quercetin glycosides dominate, though kaempferol, luteolin, and apigenin are also present, while fruits primarily contain quercetin glycosides, with only trace amounts of kaempferol and myricetin (Miean and Mohamed, 2001). Flavonoids such as quercetin, kaempferol, and apigenin in *Carica papaya* leaves exhibit significant biological activities (Nugroho et al., 2017). In chicory, kaempferol and quercetin glucuronides have been identified (Rees and Harborne, 2008), and in grape leaves, quercetin 3-O-glucoside, isorhamnetin 3-O-glucoside, and small amounts of kaempferol 3,7-O-diglycoside are present (Park and Cha, 2003).Tiliroside, a unique flavonoid identified in *Tilia miqueliana* Maxim., exhibits diverse biological activities, including anti-oxidation, anti-microbial, anti-inflammatory, anti-diabetic, and hepatoprotective effects (Grochowski et al., 2018; Matsuda et al., 2002; Goto et al., 2012; Silva et al., 2013; Luhata and Luhata, 2017; Kaur et al., 2024). Its broad therapeutic potential underscores its significance for health care. Furthermore, studies on *Camellia sinensis* reveal that light exposure significantly affects phenolic content, with total phenolic levels being higher in July compared to May or September (Soni et al., 2015).

### 4.2 Structural genes promote the accumulation of flavonoids in the leaves of *Tilia miqueliana* Maxim.

**Figure 13:**
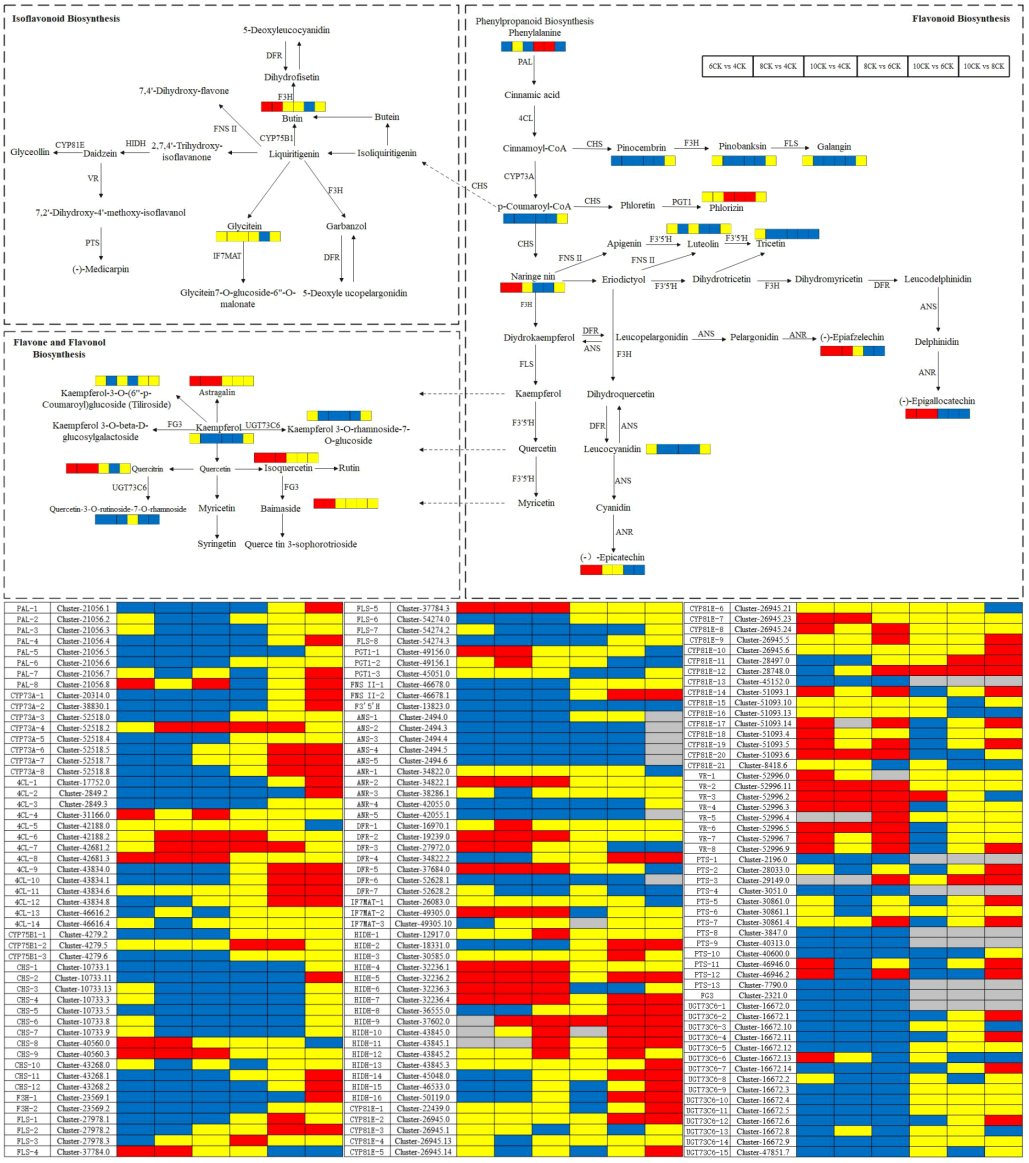
Schematic representation of flavonoid biosynthesis, isoflavonoid biosynthesis, and flavone and flavonol biosynthesis regulation with comparative analysis between distinct experimental groups. Metabolite expression levels are annotated adjacent to their corresponding metabolites within the pathway, while gene expression levels are displayed below. Red rectangles denote significant upregulation of expression, blue rectangles indicate significant downregulation, yellow rectangles represent no statistically significant change, and gray rectangles reflect data points without meaningful reference values.

Flavonoid biosynthesis begins with key precursors: phenylalanine and malonyl CoA, which are derived from the shikimic acid pathway and the TCA cycle. Phenylalanine is converted into cinnamic acid by phenylalanine ammonia lyase (PAL), which is then hydroxylated to p-coumaric acid by cinnamic acid 4-hydroxylase (C4H). P-coumaric acid is subsequently converted into 4-coumaroyl CoA by p-coumaric acid coenzyme A ligase (4CL). Several enzymes, such as FLS, F3H, and UFGT, catalyze further reactions, converting these intermediates into various flavonoid compounds (Cheng et al., 2009; Samanta et al., 2011). Among these enzymes, PAL, C4H, and 4CL play critical roles in flavonoid metabolism, with PAL and C4H showing significant correlations with many metabolites (Du et al., 2024). Studies have shown a positive correlation between quercetin levels and PAL mRNA expression, while C4H expression does not align with quercetin concentration (Cheniany and Ganjeali, 2016). In *Camellia sinensis*, the biosynthesis of lignin and flavonoids shares a key intermediate, 4CL. Overexpression of the Cs4CL2 gene in leaves significantly increases flavonoid levels (Li et al., 2022). In *Tilia miqueliana* Maxim., PAL activity is positively correlated with flavonoid content. Research has demonstrated that high expression of PAL, 4CL, and UDP-glucose-flavonoid 3-O-glucosyltransferase (UF3GT) promotes flavonoid accumulation, while high expression of flavonoid 3’-hydroxylase (F3’H) and flavonol synthase (FLS) results in the predominance of flavonols (Park et al., 2023; Lu et al., 2024). The Pearson heatmap analysis in this study revealed a strong positive correlation between flavonoids, phenolic acids, and amino acids, suggesting shared biosynthetic pathways or regulatory mechanisms. In contrast, quercetin, isorhamnetin, and luteolin exhibited weaker correlations with other compounds, implying that these metabolites may have more independent regulation and synthesis pathways. The weak correlation between luteolin and scopoletin suggests their potential independence or distinct roles within the metabolic pathway. Supporting studies have observed similar patterns. In Glycyrrhiza species, key differentially expressed genes (DEGs) involved in flavonoid biosynthesis include CYP81E, PTS, VR, IFR, CYP93B2_16, IF7MAT, and HIDH (Lu et al., 2025). During flower development in *Chrysanthemum morifolium* ‘Boju’, CYP81E contributes to the biosynthesis of flavonoids like kaempferol (Chu et al., 2024). In *Tetrastigma hemsleyanum*, genes such as CHS, CHR, and IF7MAT are key to flavonoid and isoflavonoid biosynthesis, particularly under cold stress (Liu et al., 2022). In *Sophora alopecuroides* L., structural genes like IFS, HID, IF7GT, and IF7MAT regulate isoflavonoid biosynthesis (Huang et al., 2023).The candidate DEGs related to flavonoid biosynthesis identified across the four developmental stages in *Tilia miqueliana* Maxim. leaves are consistent with those found in other plant species, suggesting a conserved flavonoid biosynthetic pathway (**Figure 13**).

## 5 Conclusion

This study used targeted metabolomic and transcriptomic analyses (UPLC-ESI-MS/MS) to identify 1,971 metabolites in *Tilia miqueliana* Maxim. leaves, including flavonoids, amino acids, phenolic acids, and terpenoids. Flavonoids, amino acids, and phenolic acids comprised nearly half of the metabolites, with key bioactive compounds like tiliroside, scopoletin, quercetin, and puerarin. Significant differences in differentially accumulated metabolites (DAMs) and differentially expressed genes (DEGs) were found across developmental stages, with the largest differences between 10CK vs 4CK and 10CK vs 6CK. Over 30,000 DEGs were identified, showing substantial differences between leaves in August and April. Quantification revealed variability in metabolite levels, with strong correlations between PAL and C4H enzymes and metabolite synthesis, indicating shared biosynthetic pathways. WGCNA and pathway analysis highlighted links between gene expression and flavonoid levels. This study provides insights into pharmacologically active compounds in *Tilia miqueliana* Maxim. leaves, aiding the selection of optimal harvest times for compound extraction.

## Data Availability

The datasets presented in this study can be found in online repositories. The names of the repository/repositories and accession number(s) can be found in the article/**Supplementary Material**.
