# Supplementary material for: Integrative transcriptomics and metabolomics reveal the biosynthesis of flavonoid metabolites in Tilia miqueliana Maxim. leaves
Source: Front Plant Sci. 2025 Sep 12;16:1642949. doi: 10.3389/fpls.2025.1642949 (PMC12463861; doi:10.3389/fpls.2025.1642949)
Supplement: Supplementary file 1 [file Image1.pdf]

Integrative transcriptomics and metabolomics reveal the biosynthesis of flavonoid metabolites in *Tilia miqueliana Maxim.* leaves

Yajing Zhou [1] ·Yongbao Shen [1,2,3,*]

1 College of Forestry and Grassland, Nanjing Forestry University, Nanjing 210037, China
2 Collaborative Innovation Center of Sustainable Forestry in Southern China, Nanjing Forestry University, Nanjing 210037, China
3 Southern Tree Seed Inspection Center, National Forestry and Grassland Administration, Nanjing 210037, China
* Author for Correspondence; e-mail: ybshen@njfu.com.cn

Figure S1. Differential metabolite analysis represented by OPLS-DA score plots: (A) 6CK vs 4CK, (B) 8CK vs 4CK, (C) 10CK vs 4CK, (D) 8CK vs 6CK, (E) 10CK vs 6CK, and (F) 10CK vs 8CK.

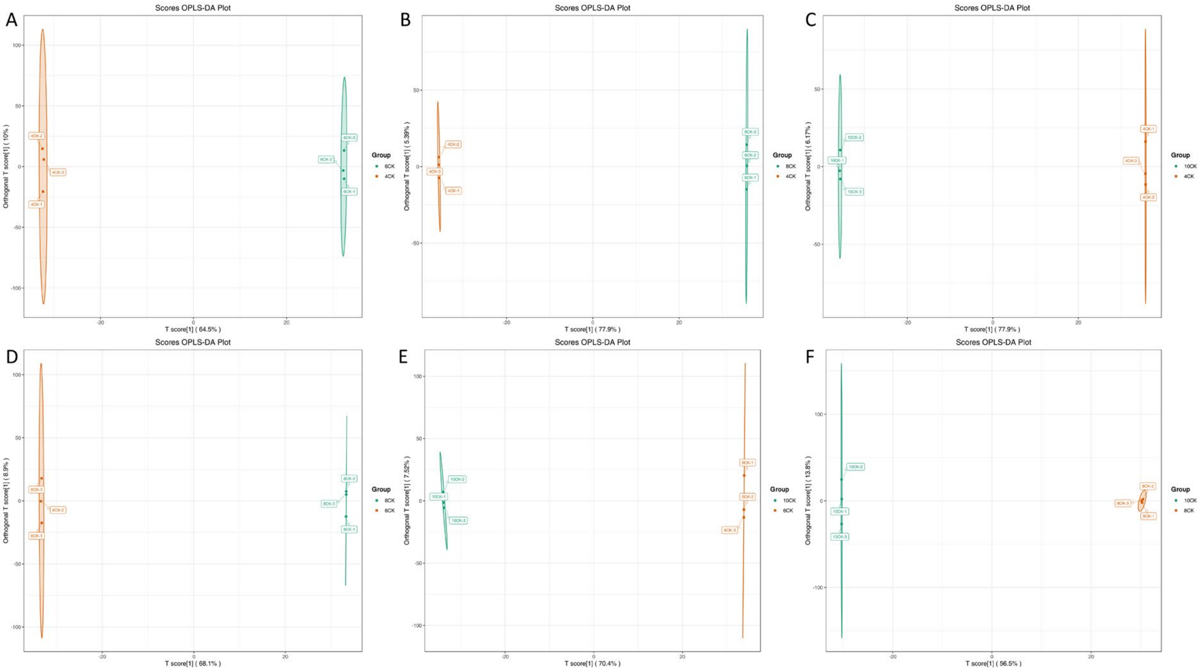

Figure S2. Volcano plots comparing differentially accumulated metabolites (DAM): (A) 6CK vs 4CK, (B) 8CK vs 4CK, (C) 10CK vs 4CK, (D) 8CK vs 6CK, (E) 10CK vs 6CK, and (F) 10CK vs 8CK.

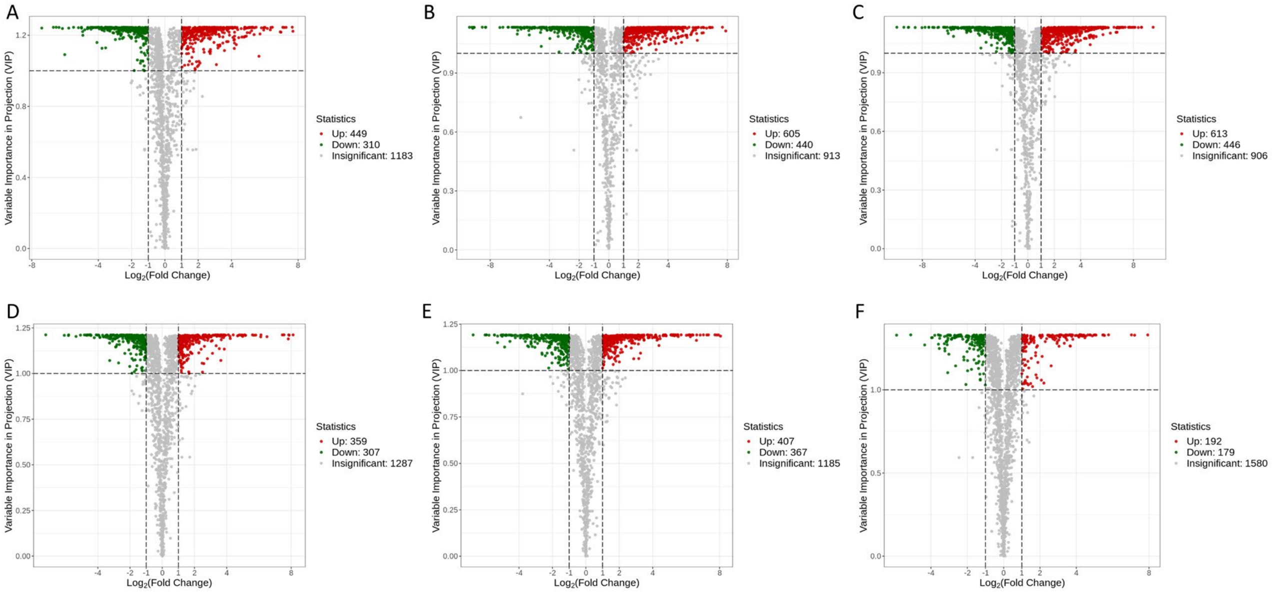

Figure S3. KEGG annotations and enrichment analysis of biomarkers in each pairwise comparison: 6CK vs 4CK (A), 8CK vs 4CK (B), 10CK vs 4CK (C), 8CK vs 6CK (D), 10CK vs 6CK (E), and 10CK vs 8CK (F).

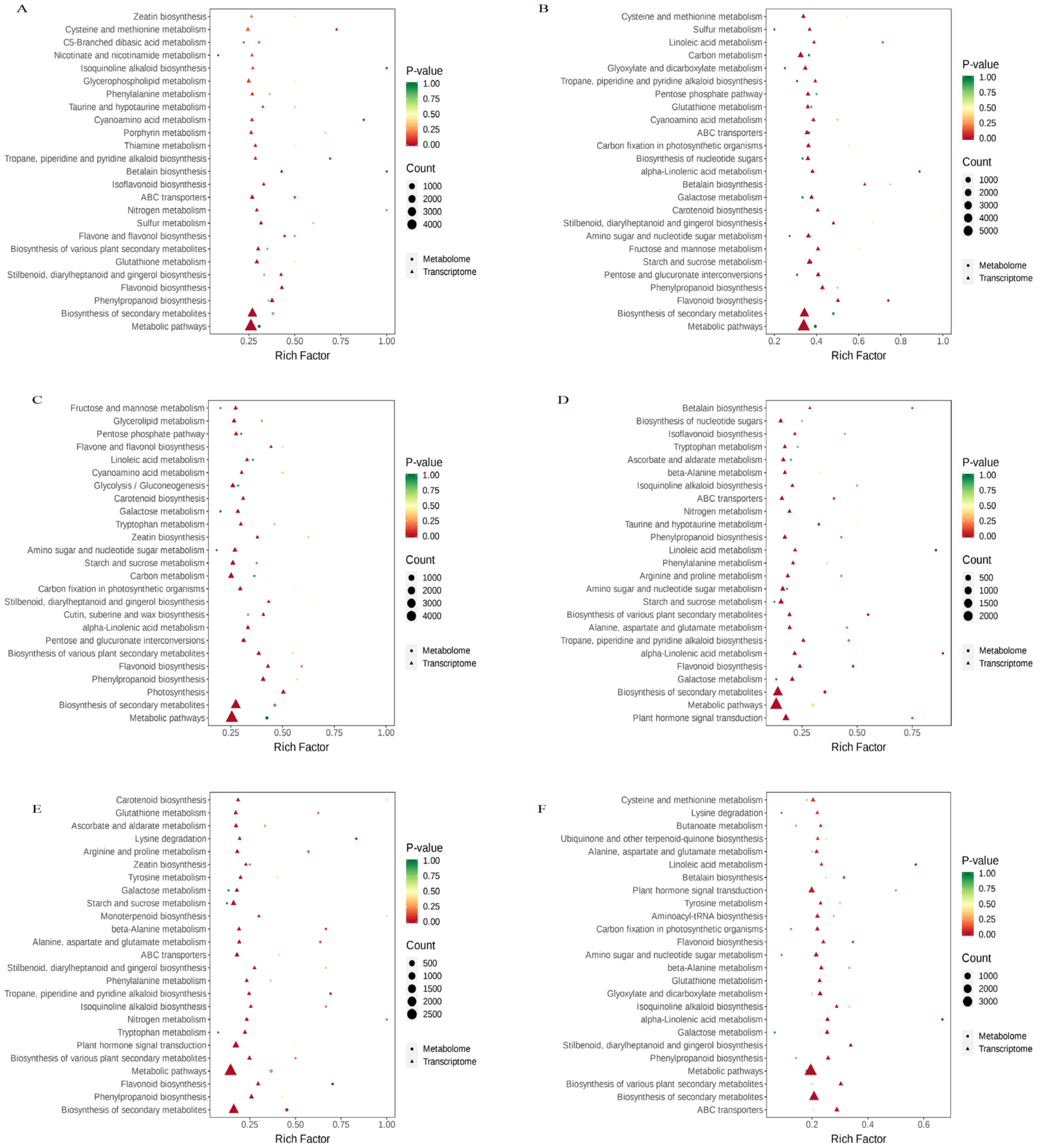

Figure S4. Relative content of key active secondary metabolites.

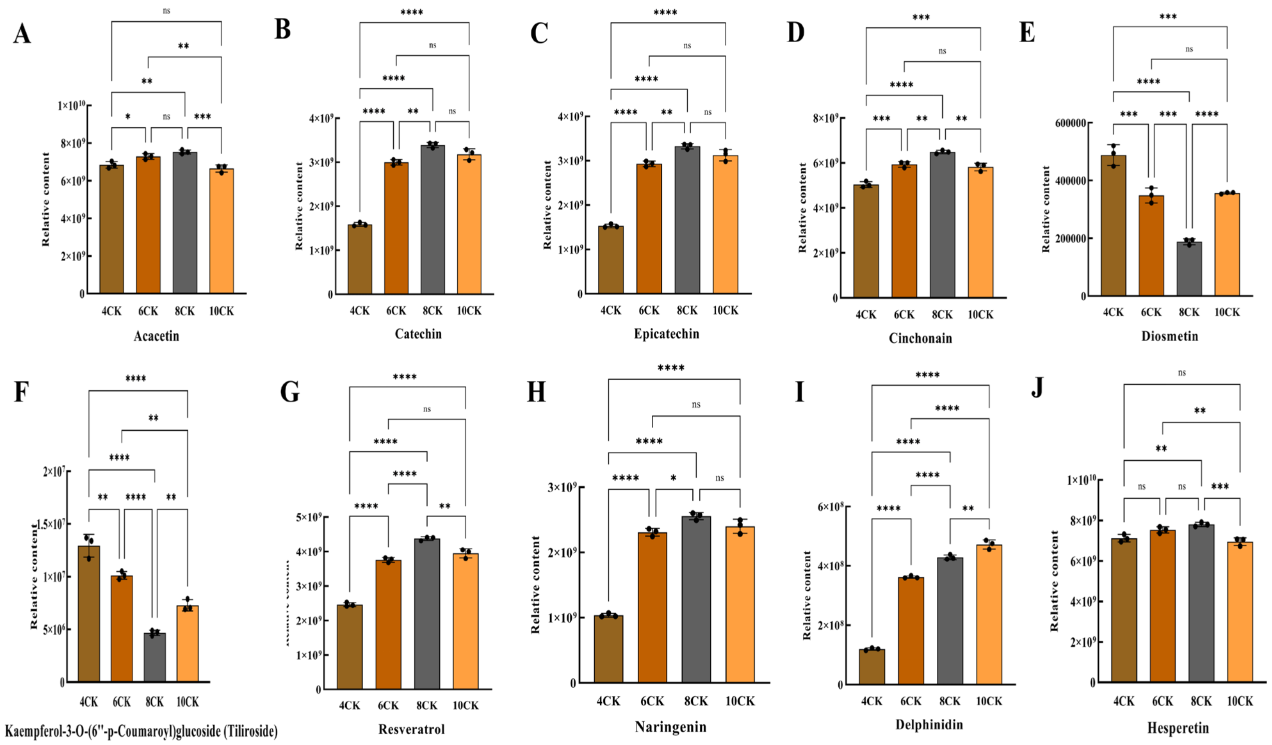

Figure S5. Relative content of other typical secondary metabolites.

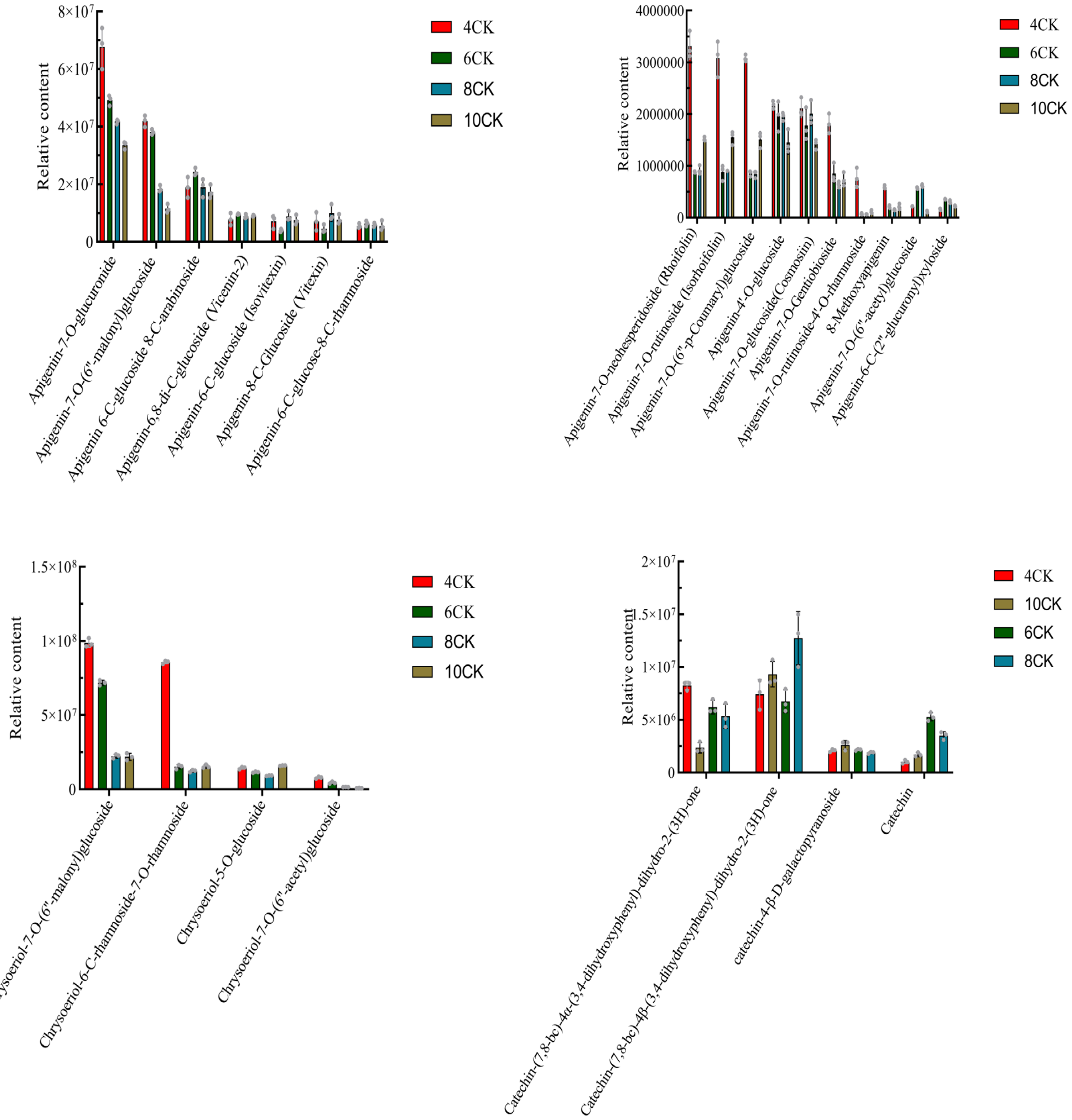

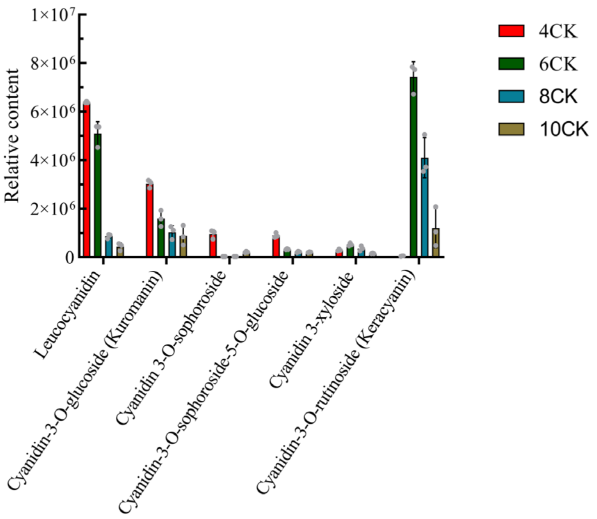

Relative content
1×10^7
8×10^6
6×10^6
4×10^6
2×10^6
0
Leucocyanidin
Cyanidin-3-O-glucoside (Kuromanin)
Cyanidin 3-O-sophoroside
Cyanidin-3-O-sophoroside-5-O-glucoside
Cyanidin 3-xyloside
Cyanidin-3-O-rutinoside (Keracyanin)
4CK
6CK
8CK
10CK

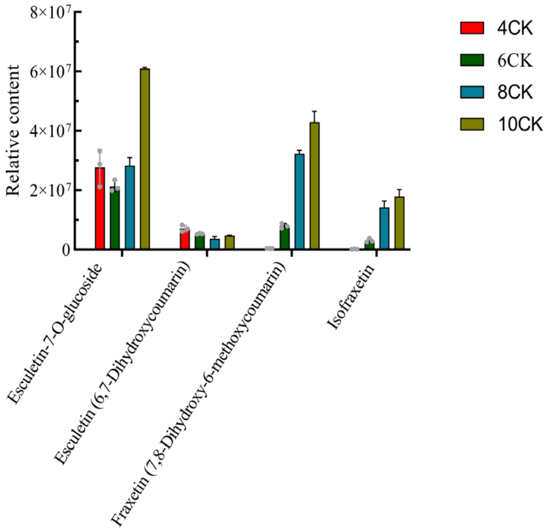

Relative content
8×10^7
6×10^7
4×10^7
2×10^7
0
Esculetin-7-O-glucoside
Esculetin (6,7-Dihydroxycoumarin)
Fraxetin (7,8-Dihydroxy-6-methoxycoumarin)
Isofraxetin
4CK
6CK
8CK
10CK

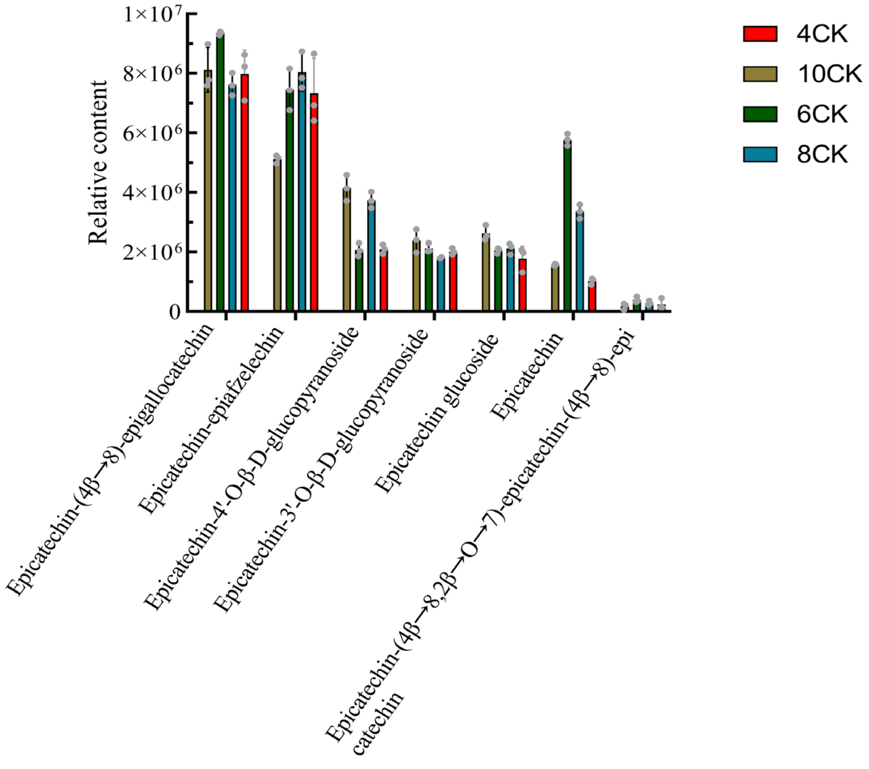

Relative content
1×10^7
8×10^6
6×10^6
4×10^6
2×10^6
0
Epicatechin-(4β→8)-epigallocatechin
Epicatechin-epiafzelechin
Epicatechin-4'-O-β-D-glucopyranoside
Epicatechin-3'-O-β-D-glucopyranoside
Epicatechin glucoside
Epicatechin
Epicatechin-(4β→8,2β→O→7)-epicatechin-(4β→8)-epi catechin
4CK
10CK
6CK
8CK

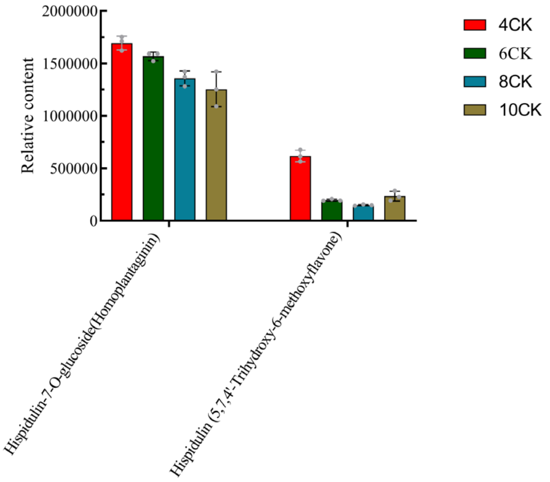
2000000
1500000
1000000
500000
0
Relative content
Hispidulin-7-O-glucoside(Homoplantaginin)
Hispidulin (5,7,4'-Trihydroxy-6-methoxyflavone)
4CK
6CK
8CK
10CK

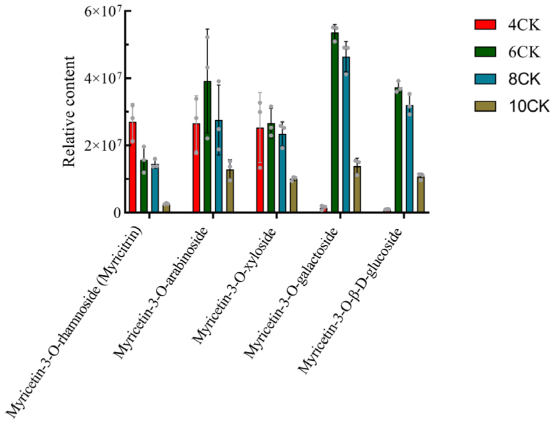
6×10⁷
4×10⁷
2×10⁷
0
Relative content
Myricetin-3-O-rhamnoside (Myricitrin)
Myricetin-3-O-arabinoside
Myricetin-3-O-xyloside
Myricetin-3-O-galactoside
Myricetin-3-O-β-D-glucoside
4CK
6CK
8CK
10CK

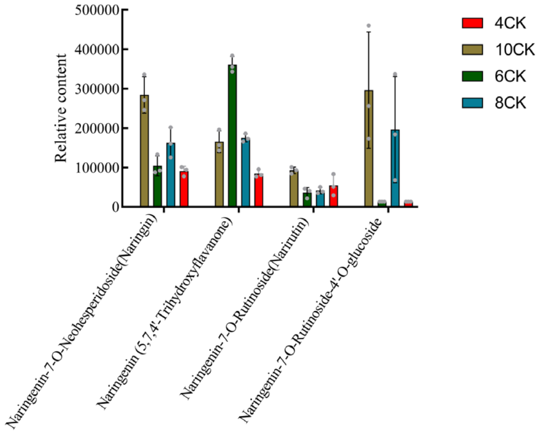
500000
400000
300000
200000
100000
0
Relative content
Naringenin-7-O-Neohesperidoside(Naringin)
Naringenin (5,7,4'-Trihydroxyflavanone)
Naringenin-7-O-Rutinoside(Narirutin)
Naringenin-7-O-Rutinoside-4'-O-glucoside
4CK
10CK
6CK
8CK

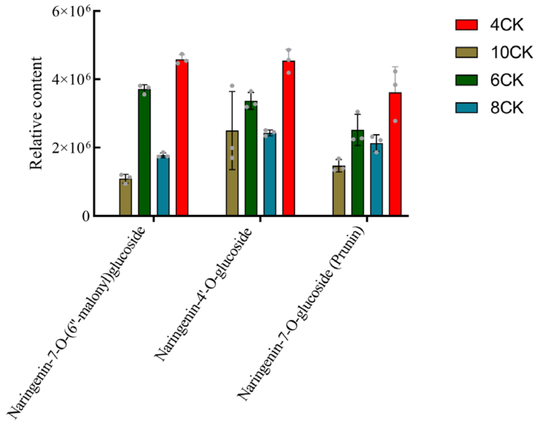
6×10⁶
4×10⁶
2×10⁶
0
Relative content
Naringenin-7-O-(6''-malonyl)glucoside
Naringenin-4'-O-glucoside
Naringenin-7-O-glucoside (Prunin)
4CK
10CK
6CK
8CK

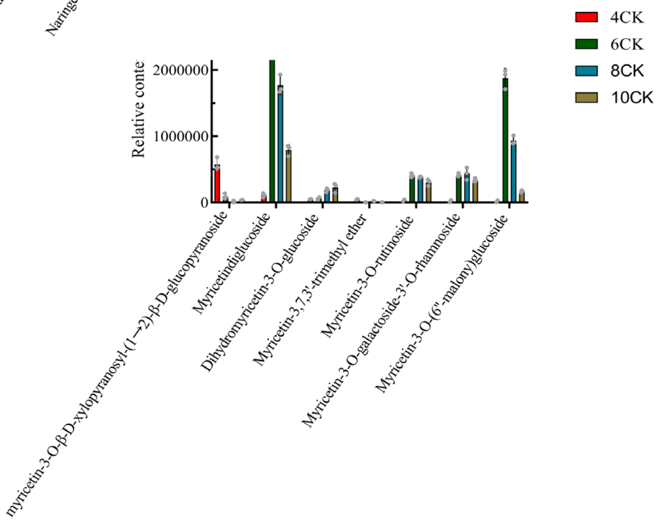
2000000
1000000
0
Relative conte
myricetin-3-O-β-D-xylopyranosyl-(1→2)-β-D-glucopyranoside
Myricetindiglucoside
Dihydromyricetin-3-O-glucoside
Myricetin-3,7,3'-trimethyl ether
Myricetin-3-O-rutinoside
Myricetin-3-O-galactoside-3'-O-rhamnoside
Myricetin-3-O-(6''-malonyl)glucoside
4CK
6CK
8CK
10CK

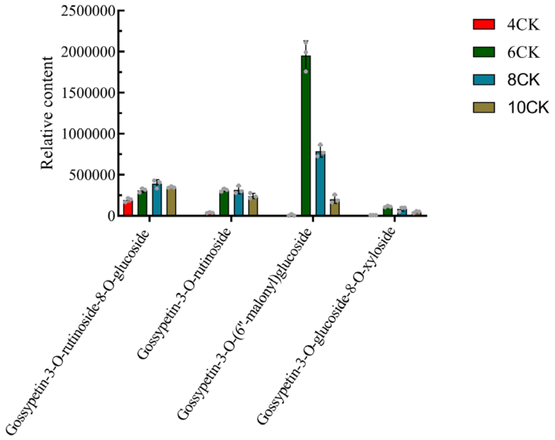
Relative content
2500000
2000000
1500000
1000000
500000
0
Gossypetin-3-O-rutinoside-8-O-glucoside
Gossypetin-3-O-rutinoside
Gossypetin-3-O-(6''-malonyl)glucoside
Gossypetin-3-O-glucoside-8-O-xyloside
4CK
6CK
8CK
10CK

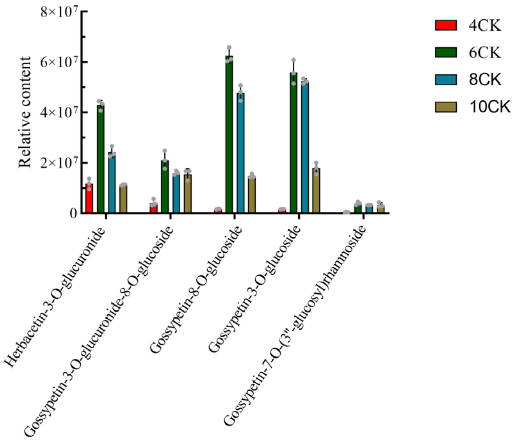
Relative content
8×10^7
6×10^7
4×10^7
2×10^7
0
Herbacetin-3-O-glucuronide
Gossypetin-3-O-glucuronide-8-O-glucoside
Gossypetin-8-O-glucoside
Gossypetin-3-O-glucoside
Gossypetin-7-O-(3''-glucosyl)rhamnoside
4CK
6CK
8CK
10CK

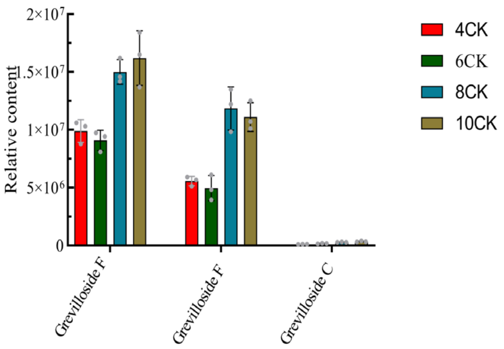
Relative content
2×10^7
1.5×10^7
1×10^7
5×10^6
0
Grevilloside F
Grevilloside F
Grevilloside C
4CK
6CK
8CK
10CK

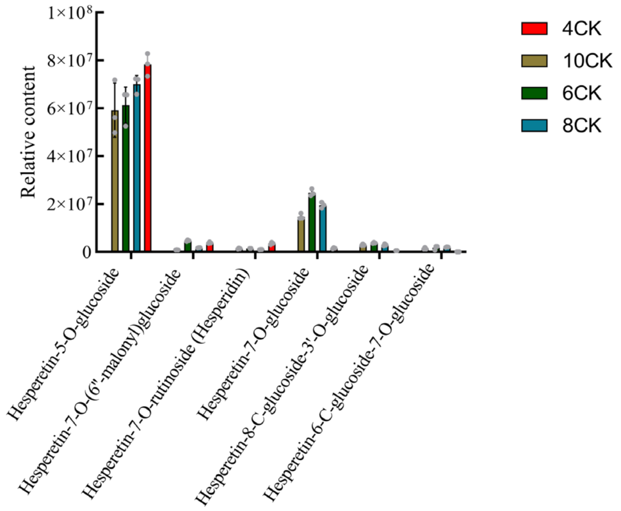
Relative content
1×10^8
8×10^7
6×10^7
4×10^7
2×10^7
0
Hesperetin-5-O-glucoside
Hesperetin-7-O-(6''-malonyl)glucoside
Hesperetin-7-O-rutinoside (Hesperidin)
Hesperetin-7-O-glucoside
Hesperetin-8-C-glucoside-3'-O-glucoside
Hesperetin-6-C-glucoside-7-O-glucoside
4CK
10CK
6CK
8CK

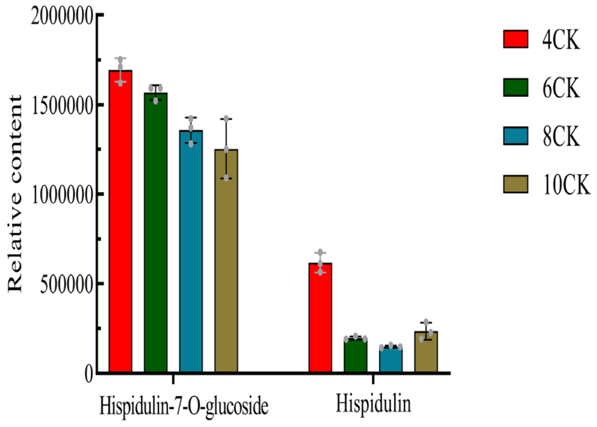
Relative content
2000000
1500000
1000000
500000
0
Hispidulin-7-O-glucoside
Hispidulin
4CK
6CK
8CK
10CK
